# Cationic Perylene Antivirals with Aqueous Solubility for Studies In Vivo

**DOI:** 10.3390/ph15101178

**Published:** 2022-09-22

**Authors:** Anna A. Shtro, Anzhelika V. Garshinina, Vera A. Alferova, Polina N. Kamzeeva, Viktor P. Volok, Ekaterina S. Kolpakova, Timofei D. Nikitin, Alexey A. Chistov, Evgeny S. Belyaev, Vladimir A. Korshun, Liubov I. Kozlovskaya, Andrey V. Aralov

**Affiliations:** 1 Smorodintsev Research Institute of Influenza, 197376 Saint Petersburg, Russia; 2 Shemyakin-Ovchinnikov Institute of Bioorganic Chemistry, Russian Academy of Sciences, 117997 Moscow, Russia; 3 Gause Institute of New Antibiotics, 119021 Moscow, Russia; 4 Chumakov Scientific Center for Research and Development of Immune-and-Biological Products, Russian Academy of Sciences (Institute of Poliomyelitis), 108819 Moscow, Russia; 5 Frumkin Institute of Physical Chemistry and Electrochemistry, Russian Academy of Science, 119071 Moscow, Russia; 6 Institute of Translational Medicine and Biotechnology, Sechenov Moscow State Medical University, 119991 Moscow, Russia

**Keywords:** perylene, broad-spectrum antivirals, in vivo protective activity, solubility, influenza

## Abstract

Perylene-based compounds are attracting significant attention due to their high broad-spectrum antiviral activity against enveloped viruses. Despite unambiguous results of in vitro studies and high selectivity index, the poor water solubility of these compounds prevented in vivo evaluation of their antiviral properties. In this work, we synthesized a series of compounds with a perylene pharmacophore bearing positively charged substituents to improve the aqueous solubility of this unique type of antivirals. Three types of charged groups were introduced: (1) quaternary morpholinium salts (**3a–b**); (2) a 2′-*O*-l-valinyl-uridine hydrochloride residue (**8**), and (**3**) a 3-methylbenzothiazolium cation (**10**). The synthesized compounds were evaluated based both on antiviral properties in vitro (CHIKV, SARS-CoV-2, and IAV) and on solubility in aqueous media. Compound 10 has the greatest aqueous solubility, making it preferable for pre-evaluation by intragastrical administration in a mouse model of lethal influenza pneumonia. The results indicate that the introduction of a positively charged group is a viable strategy for the design of drug candidates with a perylene scaffold for in vivo studies.

## 1. Introduction

Epidemics of diseases associated with various viruses, such as Influenza A (H1N1) and Ebola viruses, as well as middle east respiratory syndrome coronavirus (MERS-CoV) and severe acute respiratory syndrome coronaviruses 1 and 2 (SARS-CoV-1 and 2) occurred in 2009, 2014, 2012, 2003 and 2019, respectively. Re-emerging EBOVs and new H1N1, MERS, and SARS viruses pose a great threat to humanity and easily cross country borders. The traditional “one-bug – one drug” approach is insufficient for addressing the issue of re-emerging and new viral pathogens, and few drugs are currently available to control epidemic viral diseases [1,2,3,4,5]. Thus, it is critical to develop a class of broad-spectrum antiviral agents.

Antivirals with broad-spectrum activity can be divided into two categories: direct-acting drugs and host-targeting antivirals that engage host-cell machinery important for maintaining various stages of the viral life cycle. Representatives of the former class usually demonstrate an option for repurposing, high selectivity, and low cytotoxicity. Direct-acting antivirals include inhibitors of virus attachment (broadly neutralizing antibodies (bNAbs) [6,7] and sialidase fusion protein DAS181 [8]), virus entry (small molecules 5705213/7402683 [9] and the peptide enfuvirtide [10]), replication (galidesivir [11], favipiravir [12] ribavirin [13], and remdesivir [14,15]), viral assembly and budding (verdinexor [16] and FGI-104 [17]). Among them, replication inhibitors, the most promising candidates for the development of optimal broad-spectrum agents, still exhibit a number of disadvantages such as low plasma concentrations resulting from rapid renal elimination for favipiravir [18,19,20], poor selectivity and toxicity leading to undesirable side effects for ribavirin [21], and poor oral bioavailability and short half-life for remdesivir [22]. As a separate prospective subclass, yet affecting only enveloped viruses, agents targeting the viral envelope (LJ001 [23] and JL118/JL122 [24], derivatives of aromatic methyldiene rhodanine and oxazolidine-2,4-dithione, respectively) can be distinguished.

Enveloped viruses are a large class of pathogens responsible for multiple serious diseases. The membrane of the viral particle and its rearrangements during the replicative cycle appear to be a promising target for the development of broad-spectrum antiviral agents [25]. Moreover, the viral envelope is significantly more vulnerable than the mammalian cellular membrane because the virus possesses no membrane repair systems. Additionally, targeting the viral envelope is attractive due to the anticipated rate of resistance development being negligible. The viral lipid envelope originates from the host cell’s membrane and is thus not affected by natural viral diversity and variability [26].

One of the most potent classes of viral fusion inhibitors with a broad spectrum of activity against enveloped viruses are so-called rigid amphipathic fusion inhibitors (RAFIs) [27]. These compounds contain a perylene moiety and were initially discovered as nucleoside analogs. However, further studies revealed that their structures could be significantly simplified, and neither the nucleobase nor the carbohydrate moiety were vital for their antiviral action [28]. Thorough structural studies led to the development of perylene-based compounds with remarkable subnanomolar in vitro activity against a wide range of enveloped viruses [29]. The mechanism of viral fusion inhibition by perylene derivatives is not yet completely understood. There are two hypotheses, with consensus on the target – the outer lipid membrane of the virion. The first hypothesis implies intercalation of the drug into the virion lipid membrane, leading to mechanical disturbance of its rheological properties [8]. Alternatively, drug-mediated photoinduced generation of singlet oxygen, causing oxidation of unsaturated lipids in the virion membrane, was proposed [30]. Taking into account the rather controversial results of mechanistic studies, perylene derivatives presumably inhibit viral fusion in a structure-dependent combination of these two modes of action.

Despite outstanding in vitro activities, RAFIs have not been tested in vivo. The most challenging problem is the low aqueous solubility of perylene-based compounds. In this work, we aimed to improve the solubility of RAFI by introducing various positively charged moieties. We synthesized a concise series of novel perylene derivatives and assessed their antiviral properties, cytotoxicity, and solubility in aqueous media. Dramatically improved water solubility allowed studying the protective properties of the most promising compound against the influenza virus in mice.

## 2. Results and Discussion

### 2.1. Chemistry

**Scheme 1:**
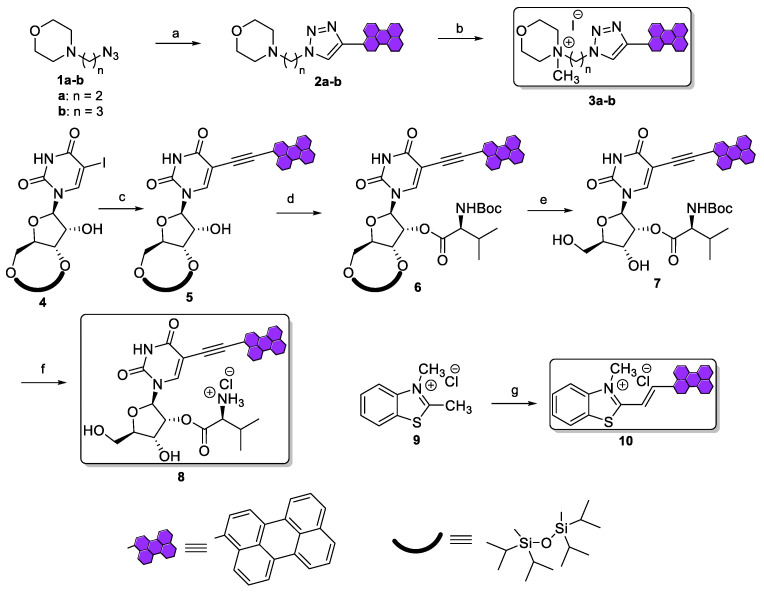
Synthetic route to positively charged perylene-based derivatives. Reagents and conditions: (**a**) 3-ethynylperylene, TBTA, CuI, DMSO, rt; (**b**) methyl iodide, CH_2_Cl_2_, 35 °C; (**c**) 3-ethynylperylene, CuI, Pd(PPh_3_)_4_, TEA, DMF, rt; (**d**) N-Boc-l-valine, DCC, DMAP, CH_2_Cl_2_, rt; (**e**) TBAF·3H_2_O, THF, rt; (**f**) HCl, 1,4-dioxane-CH_2_Cl_2_, rt; (**g**) perylene-3-carbaldehyde, Ac_2_O, reflux.

The synthesis of soluble positively charged perylene-based analogs was carried out using a number of biocompatible moieties bearing a positive charge, such as N-methylmorpholinium [31], O-l-valinyl ester hydrochloride [32], and benzothiazolium [33] residues, and introduced them via well-established synthetic procedures (Scheme 1).

First, N-(ω-azidoalkyl)morpholine **1** was condensed with 3-ethynylperylene under Cu(I)-catalyzed azide-alkyne cycloaddition (CuAAC) conditions [28]. Then, the N atom of the morpholine ring was alkylated with methyl iodide, affording target positively charged derivatives **3a** and **3b**, with an ethyl (n = 2) or propyl (n = 3) bridge, respectively. Nucleoside derivative **8** was prepared in four steps. 3′,5′-*O*-silyl protected 5-iodouridine **4 [34]** was subjected to Sonogashira coupling with 3-ethynylperylene, yielding **5**. Condensation of **5** with *N*-Boc-l-valine using DCC as the condensing agent afforded **6**, which was then subjected to deblocking of 3′- and 5′-hydroxyls groups by treatment with TBAF·3H_2_O. The Boc protecting group from **7** was removed by hydrolysis in acidic conditions, yielding positively charged nucleoside derivative **8**. Finally, 2,3-dimethylbenzo[*d*]thiazol-3-ium chloride **9** and perylene-3-carbaldehyde, as the carbanion-forming and the carbonyl-containing electrophilic components, respectively, were subjected to a Knoevenagel condensation reaction [35] by refluxing in acetic anhydride to give final compound **10**.

NMR spectroscopy and HRMS spectrometry were used to confirm the structure and elemental composition of intermediates and target compounds (see Experimental and Figures S1–S9). The purity of target positively charged derivatives **3a–b**, **8**, and **10** was >90%, as determined by HPLC (Figures S11–S14).

### 2.2. In Vitro Activity, Aqueous Solubility, and Serum Stability Assessment

Since determining the scope of applicability of perylene-based compounds as antivirals was the main purpose of this study, we first evaluated in vitro antiviral activity (Table 1) of target compounds **3a–b**, **8**, **10** and synthetic intermediates **2a–b**, and **5–7** against a number of enveloped RNA viruses pathogenic for humans: Chikungunya virus (CHIKV), severe acute respiratory syndrome-related coronavirus 2 (SARS-CoV-2), and influenza A virus (IAV).

Charged compounds retained antiviral activity in the low micromolar-submicromolar concentration range against CHIKV and SARS-CoV-2 with almost no evidence of cytotoxicity. At the same time, some uncharged intermediates did not demonstrate any activity against CHIKV (**2b**, **6**) or SARS-CoV-2 (**2b**, **5**, **6**) in concentrations studied. In particular, replacement of the ethyl linker between perylenyltriazolyl and morpholine residues with a propyl one led to complete loss of activity against CHIKV and SARS-CoV-2 in the morpholine series, but the corresponding N-methylated derivative **3b** had activity against these two viruses. Surprisingly, 5′,3′-*O*-protected 5-(perylen-3-ylethynyl)uridine derivative **5** did not show any signs of efficacy against SARS-CoV-2, and the introduction of an additional lipophilic substituent at the 2′-OH function of the ribose residue also caused a complete loss of activity of **6** against CHIKV. Meanwhile, removal of the siloxane 5′,3′-*O*-protective group restored the activity of 7, suggesting the importance of maintaining the lipophilic perylene residue and polar, in our case hydroxy, groups, for maximum activity. Activity against IAV was substantially lower compared to CHIKV and SARS-CoV-2, surprisingly decreasing with the introduction of the positive charge in the morpholine series (**3a–b** vs. **2a–b**). In contrast, positively charged nucleoside derivative **8** has slightly better activity compared to its non-charged intermediates **6** and **7**. Finally, all charged derivatives showed comparable-to-higher activity against CHIKV and SARS-CoV-2 vs. positive control N-hydroxycytidine (NHC) [36]. All compounds had lower activity against IAV vs. Tamiflu as the control antiviral.

Then, an assessment of aqueous solubility of the positively charged compounds was performed to select a candidate for studies in a model of lethal influenza pneumonia in mice (Table 2).

Positively charged derivatives **3a–b** and **10** were found to have substantial solubility in water, whereas positively charged and uncharged nucleoside analogs **8** and **aUY11** had poor and no solubility, respectively. Among the candidates synthesized, **8** had the highest antiviral activity against influenza A virus; however, its low aqueous solubility limits evaluation in vivo. Since, among the remaining three derivatives, compound **10** had the highest potency and solubility, it was chosen for subsequent evaluation in a mouse model of influenza A virus infection.

Next, human intestinal absorption (cHIA) [38] and blood–brain barrier permeability (clogBB) [39] were estimated using predictive QSAR models [40]. Most of the compounds were able to penetrate the blood–brain barrier effectively, and derivative **10** demonstrated the highest level of intestinal absorption, and even distribution between the brain and blood, which may be useful in the treatment of various brain lesion complications associated with influenza (Table S1) [41,42].

Since in vivo evaluation of the leader compound in mouse model involves intragastric administration, stability in stomach acid-mimicking 0.16 M aqueous HCl was assessed. The compound was stable, with slight signs of decomposition as determined by thin-layer chromatography (TLC) for at least 4 h, which exceeds the average residence time of drugs in the stomach [43]. Then, stability in fetal bovine serum (FBS) was evaluated. In vitro FBS half-life (t_1/2_) was found to be 167 min (Figure S10), which is comparable to approved drugs and other drug candidates [44,45,46].

### 2.3. In Vivo Studies

**Figure 1:**
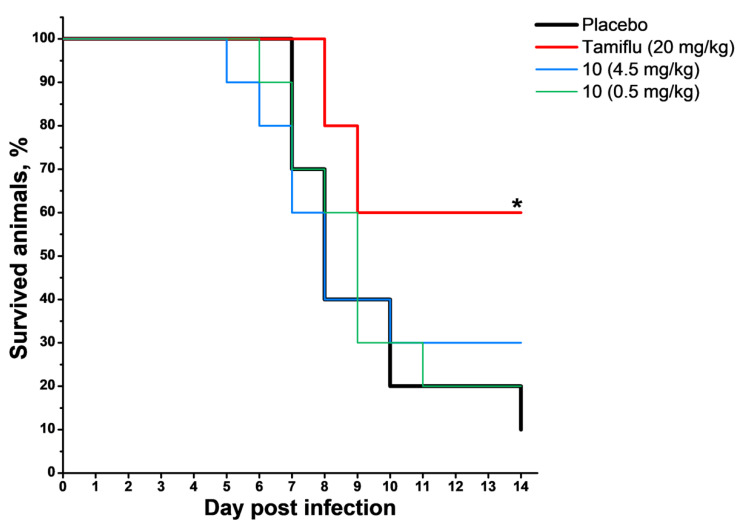
Kaplan–Meier survival curves during experimental influenza pneumonia in BALB/c mice upon infection with influenza A/California/7/09/MA virus (N = 10); *—difference between Placebo and Tamiflu groups is statistically significant (*p* = 0.02084).

**Figure 2:**
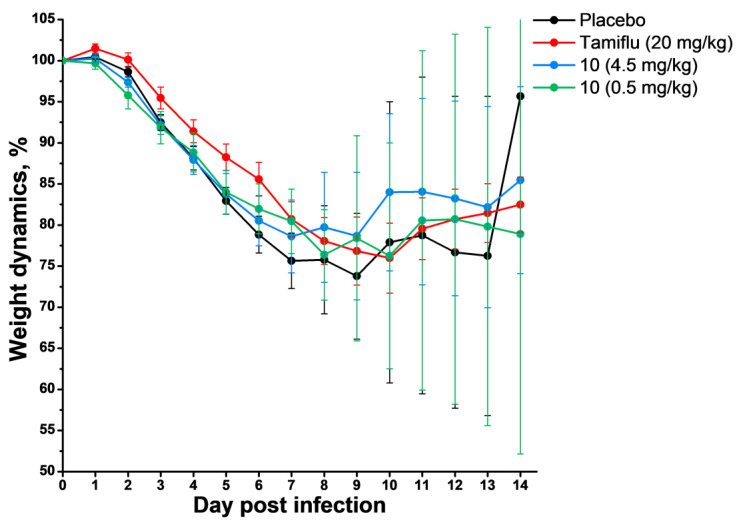
Dynamics of changes in body weight of BALB/c mice with experimental influenza pneumonia upon infection with influenza A/California/7/09/MA virus (N = 10).

Protective efficacy for compound **10** was assessed by the ability to prevent the development of lethal influenza pneumonia in mice. BALB/c mice were treated intragastrically with the compound in two different dosing regimens, placebo and comparative drug Tamiflu [47,48] for the period between one day prior and three days after intranasal infection with IAV. Clinical symptom development and body weight were observed daily. Survival rates and curves are presented in Table 3 and Figure 1, respectively, and body weight changes in Figure 2.

Infection with the IAV strain A/California/7/09/MA caused mouse mortality starting from day 5 post infection (p.i.). By the end of the experiment, mortality was 90% in the Placebo group and 40% (*p* = 0.015) in the comparison group (Tamiflu), which corresponded to a protection index (PI) of 56% and was consistent with available information for this strain [49]. The use of compound **10** in a dose of 0.5 or 4.5 mg/kg did not cause a significant reduction in the mortality rate compared to the Placebo group (*p* > 0.05).

Influenza virus infection induced a reduction in body weight of all animals in all groups, reaching a maximum of 26.2% on day 9 p.i. and returning to the initial values on day 14 p.i. in the Placebo group. In the comparison group (Tamiflu), the reduction was less pronounced and reached 24.0% on day 10 p.i. The experimental group treated with 4.5 mg/kg of **10** showed less weight loss (up to 21.3% on day 9 p.i.) and faster weight recovery compared to the Placebo group.

To conclude, mice treated with **10** (4.5 mg/kg) showed a lower mortality rate and faster weight recovery, although the differences were not significant.

## 3. Materials and Methods

### 3.1. General Information

All solvents and reagents were commercially available and used without purification. Spots on silica gel-precoated TLC plates (60 μm, F_254_, Merck, Germany) were visualized using UV light (254 and 365 nm), and column chromatography was performed on silica gel (0.040−0.063 mm, Merck, Germany). ^1^H and ^13^C NMR spectra were recorded on a Bruker Avance III 600 spectrometer (Bruker BioSpin, Germany) at 600 and 150 MHz, respectively. DMSO-*d*_6_ (2.50/39.52 ppm for ^1^H and ^13^C spectra, respectively) was used as the internal standard. Signals were described as follows: chemical shift (multiplicity (s = singlet, d = doublet, t = triplet, m = multiplet), coupling constant(s) (Hz), number of protons) for ^1^H spectra, and chemical shift (number of carbons) for ^13^C spectra. HRMS spectra were registered in the positive ion mode on a Thermo Scientific LTQ Orbitrap hybrid instrument (Thermo Electron Corp., Bremen, Germany) with direct sample infusion. HPLC and LCMS analysis was performed using an Agilent 1260 Infinity II instrument (with a single quadrupole 6125B mass detector), equipped with a ZORBAX RRHT SB-C18, 2.1 × 50 mm, 1.8 μm column. The column was eluted with a linear gradient (5–95% in 10 min) of LCMS-grade acetonitrile (with 0.1 vol. % formic acid) in LCMS-grade water (with 0.1 vol. % formic acid) at a 0.4 mL/min flow rate. 4-(2-Azidoethyl)morpholine **1a**, 4-(3-azidopropyl)morpholine **1b**, 1-[3′,5′-*O*-(tetraisopropyldisiloxan-1,3-diyl)-β-d-ribofuranosyl]-5-iodouracil **4** and 3-ethynylperylene were synthesized according to literature [28,34,50]. An aqueous solution of 2,3-dimethylbenzo[*d*]thiazol-3-ium iodide [51] was eluted through a column filled with Bio-Rad AG^®^ MP-1M Anion Exchange Resin (chloride form, 10 eq.) to afford 2,3-dimethylbenzo[*d*]thiazol-3-ium chloride.

### 3.2. Synthesis

#### 3.2.1. General Procedure for CuAAC Reaction

The corresponding azido-containing morpholine derivative **1** (0.5 mmol), 3-ethynylperylene (138 mg, 0.5 mmol), TBTA (14 mg, 0.025 mmol), CuI (5 mg, 0.025 mmol), and DMSO (5 mL) were mixed, degassed, and then stirred for 16 h at room temperature under argon atmosphere. To the reaction solution, water (15 mL) was added, and the precipitate formed was filtered. The filter cake was dissolved in ethyl acetate (30 mL) and sequentially washed with 0.1 M aqueous EDTA solution (10 mL) and brine (30 mL). The organic layer was dried over Na_2_SO_4_ and concentrated in vacuo. The crude product was purified by column chromatography on silica gel (0–2% MeOH in CH_2_Cl_2_), yielding the corresponding product.

##### 4-(2-(4-(Perylen-3-yl)-1H-1,2,3-triazol-1-yl)ethyl)morpholine **2a**

Yield 190 mg (88%); amorphous brown solid. ^1^H NMR (600 MHz, DMSO-*d_6_*): δ 8.62 (s, 1H), 8.46–8.37 (m, 5H), 7.84–7.77 (m, 3H), 7.63–7.53 (m, 3H), 4.62 (t, J = 6.2 Hz, 2H), 3.60–3.55 (m, 4H), 2.87 (t, J = 6.2 Hz, 2H), 2.50–2.46 (m, 4H, overlapping with DMSO). ^13^C NMR (150 MHz, DMSO-*d_6_*): δ 145.0, 134.0, 131.4, 130.6, 130.3 (2C), 130.0, 128.2, 127.9, 127.8, 127.7, 127.5, 127.4, 127.1, 126.8, 126.7, 125.3, 124.5, 120.8, 120.8, 120.7, 120.3, 66.0 (2C), 57.1, 52.8 (2C), 46.5. HRMS (ESI) m/z: calcd for C_28_H_25_N_4_O^+^ [M + H]^+^: 433.2023; found 433.2020.

##### 4-(3-(4-(Perylen-3-yl)-1H-1,2,3-triazol-1-yl)propyl)morpholine **2b**

Yield 192 mg (86%); amorphous brown solid. ^1^H NMR (600 MHz, DMSO-*d_6_*): δ 8.65 (s, 1H), 8.49–8.34 (m, 5H), 7.86–7.75 (m, 3H), 7.66–7.51 (m, 3H), 4.58–4.46 (m, 2H), 3.65–3.52 (m, 4H), 2.43–2.30 (m, 6H), 2.17–2.06 (m, 2H). ^13^C NMR (150 MHz, DMSO-*d_6_*): δ 145.3, 134.1, 131.4, 130.6, 130.4, 130.4, 130.1, 128.3, 128.0, 127.9, 127.7, 127.6, 127.5, 127.2, 126.9, 126.9, 125.5, 124.2, 120.9, 120.9, 120.8, 120.4, 66.0 (2C), 54.8, 53.1 (2C), 47.8, 26.5. HRMS (ESI) m/z: calcd for C_29_H_27_N_4_O^+^ [M + H]^+^: 447.2179; found 447.2173.

#### 3.2.2. General Procedure for Methylation with Methyl Iodide

To a solution of **2** (0.3 mmol) in CH_2_Cl_2_ (30 mL), methyl iodide (0.62 mL, 3 mmol) was added in one portion, and the reaction mixture was stirred at 35 °C overnight. The precipitate that formed was filtered, sequentially washed with Et_2_O (3 × 10 mL), CH_2_Cl_2_ (3 × 10 mL), and 10% MeOH in CH_2_Cl_2_ (10 mL, *v*/*v*), dried over Na_2_SO_4,_ and concentrated in vacuo, yielding the corresponding methylated product.

##### 4-Methyl-4-(2-(4-(perylen-3-yl)-1H-1,2,3-triazol-1-yl)ethyl)morpholin-4-ium iodide **3a**

Yield 122 mg (71%); amorphous brown solid. HPLC rt 7.5 min (Figure S11). ^1^H NMR (600 MHz, DMSO-*d_6_*): δ 9.24 (s, 1H), 8.60 (d, J = 7.9 Hz, 1H), 8.60 (d, J = 7.9 Hz, 1H), 8.55 (t, J = 7.8 Hz, 1H), 8.49 (d, J = 7.4 Hz, 1H), 7.94 (d, J = 8.1 Hz, 1H), 7.91 (d, J = 8.1 Hz, 1H), 7.87 (d, J = 7.8 Hz, 1H), 7.69–7.61 (m, 3H), 7.57 (d, J = 8.2 Hz, 1H), 4.91–4.86 (m, 2H), 4.17 (s, 3H), 3.63–3.58 (m, 4H), 2.99–2.93 (m, 2H), 2.56–2.52 (m, 4H). ^13^C NMR (150 MHz, DMSO-*d_6_*): δ 139.7, 134.2, 134.0, 132.5, 131.3, 131.1, 130.7, 129.6, 129.4, 129.2, 128.7, 128.4, 128.0, 127.5, 127.1, 127.0, 123.9, 122.3, 121.8, 121.5, 119.9, 118.6, 66.1 (2C), 55.9, 52.6 (2C), 50.3, 38.1. HRMS (ESI) m/z: calcd for C_29_H_27_N_4_O^+^ [M-I]^+^: 447.2179; found 447.2182.

##### 4-Methyl-4-(3-(4-(Perylen-3-yl)-1H-1,2,3-triazol-1-yl)propyl)morpholin-4-ium iodide **3b**

Yield 115 mg (65%); amorphous brown solid. HPLC rt 7.5 min (Figure S12). ^1^H NMR (600 MHz, DMSO-*d_6_*): δ 8.73 (s, 1H), 8.49–8.45 (m, 3H), 8.44–8.40 (m, 2H), 7.86–7.81 (m, 3H), 7.64 (t, J = 8 Hz, 1H), 7.60–7.56 (m, 2H), 4.62 (t, J = 6.8 Hz, 2H), 3.98–3.91 (m, 4H), 3.64–3.58 (m, 2H), 3.52–3.46 (m, 4H), 3.18 (s, 3H), 2.50–2.44 (m, 2H). ^13^C NMR (150 MHz, DMSO-*d_6_*): δ 145.5, 134.1, 131.4, 130.7, 130.6, 130.4, 130.1, 128.3, 128.1, 128.0, 127.6 (2C), 127.5, 127.2, 126.9, 126.9, 125.5, 124.3, 121.0 (2C), 120.9, 120.4, 59.7 (2C), 59.0 (2C), 48.5, 46.6, 39.4, 22.0. HRMS (ESI) m/z: calcd for C_30_H_29_N_4_O^+^ [M-I]^+^: 461.2336; found 461.2345.

#### 3.2.3. 3′,5′-*O*-(Tetraisopropyldisiloxan-1,3-diyl)-5-(perylen-3-ylethynyl)uridine **5**

A mixture of 3′,5′-*O*-(tetraisopropyldisiloxane-1,3-diyl)-5-iodouridine **4** (0.74 g, 1.2 mmol), 3-ethynylperylene (0.42 g, 1.5 mmol), Pd(PPh_3_)_4_ (0.14 g, 0.12 mmol) and CuI (0.05 g, 0.24 mmol) in DMF (20 mL) was evacuated, and then Et_3_N (0.34 mL, 2.4 mmol) was added under argon atmosphere. The reaction mixture was stirred at room temperature in the dark overnight and then partitioned between EtOAc (200 mL) and water (100 mL). The organic layer was sequentially washed with a 5% aqueous solution of citric acid (20 mL), a 1% EDTA solution (20 mL) and brine (100 mL) and evaporated in vacuo. The resulting brown oil was purified by column chromatography on silica gel (0→50% EtOAc in CH_2_Cl_2_), yielding **5** (0.76 g, 83%) as an orange foam. ^1^H NMR (600 MHz, DMSO-*d_6_*): δ 11.85 (br s, 1H), 8.45 (d, J = 7.6 Hz, 1H), 8.42–8.38 (m, 2H), 8.37 (d, J = 8.0 Hz, 1H), 8.26 (d, J = 8.2 Hz, 1H), 7.85–7.80 (m, 3H), 7.68–7.64 (m, 2H), 7.57 (t, J = 7.8 Hz, 2H), 6.11 (d, J = 7.8 Hz, 1H), 5.94 (d, J = 6.0 Hz, 1H), 4.41–4.36 (m, 1H), 4.17–4.12 (m, 1H), 4.07 (dd, J = 13.1 Hz, J = 2.0 Hz, 1H), 3.94 (dd, J = 13.1 Hz, J = 2.1 Hz, 1H), 3.77–3.73 (m, 1H), 1.08–0.89 (m, 28H). ^13^C NMR (150 MHz, DMSO-*d_6_*): δ 161.3, 149.3, 143.5, 134.1, 133.8, 131.2, 130.9, 130.3, 130.0, 129.7, 128.6, 128.2, 127.7 (2C), 127.5, 126.9, 126.8, 125.6, 121.6, 121.3, 121.2, 120.2, 119.2, 97.7, 90.7, 88.0, 82.9, 79.1, 74.3, 74.2, 59.9, 17.1 (2C), 17.1, 17.0, 16.8, 16.7 (2C), 16.6, 12.8, 12.3, 12.0, 11.8. HRMS (ESI) m/z: calcd for C_43_H_49_N_2_O_7_Si_2_^+^ [M + H]^+^: 761.3073; found 761.3036.

#### 3.2.4. 2′-*O*-(N-Boc-l-valinyl)-3′,5′-*O*-(tetraisopropyldisiloxane-1,3-diyl)-5-(perylen-3-ylethynyl)uridine **6**

To a solution of **5** (0.71 g, 0.93 mmol) and Boc-l-valine (0.61 g, 2.79 mmol) in dry CH_2_Cl_2_ (50 mL) 4-dimethylaminopyridine (DMAP) (0.01 g, 0.08 mmol) was added followed by N,N’-dicyclohexylcarbodiimide (DCC) (0.77 g, 3.72 mmol). The reaction mixture was stirred at room temperature for 4 h, washed with 5% solution of citric acid (20 mL), and concentrated in vacuo. The residue was purified by column chromatography on silica gel (50→100% CH_2_Cl_2_ in hexane), yielding **6** (0.77 g, 87%) as an orange foam. ^1^H NMR (600 MHz, DMSO-*d_6_*): δ 8.46 (br s, 1H), 8.36 (d, J = 8.2Hz, 1H), 8.24 (d, J = 7.4Hz, 1H), 8.21 (d, J = 7.6Hz, 1H), 8.19 (d, J = 7.6Hz, 1H), 8.13 (d, J = 7.9Hz, 1H), 7.87 (s, 1H), 7.72–7.67 (m, 3H), 7.61 (dd, J = 7.4 Hz, J = 8.2Hz, 1H), 7.51–7.47 (m, 2H), 6.28 (d, J = 4.7 Hz, 1H), 5.65–5.59 (m, 1H), 4.90 (d, J = 9.3 Hz, 1H), 4.60–4.51 (m, 1H), 4.30 (dd, J = 9.3 Hz, J = 3.8 Hz, 1H), 4.15 (dd, J = 3.7 Hz, J = 12.8 Hz, 1H), 4.10 (dd, J = 3.0 Hz, J = 12.8 Hz, 1H), 3.90–3.86 (m, 1H), 2.13–2.04 (m, 1H), 1.43 (s, 9H), 1.13–1.01 (m, 28H), 0.96 (d, J = 6.8 Hz, 3H), 0.81 (d, J = 6.8 Hz, 3H). ^13^C NMR (150 MHz, DMSO-*d_6_*): δ 171.2, 160.6, 155.4, 148.6, 141.8, 134.6, 134.6, 132.2, 131.3, 131.0, 130.8, 130.7, 128.4 (2C), 128.0, 127.5 (2C), 126.6, 126.5, 126.4, 120.9, 120.8, 120.6, 119.6, 119.4, 100.7, 93.2, 85.7, 81.4, 79.9, 77.0 (1C overlaps with CDCl_3_), 73.0 (2C), 60.7, 58.2, 30.7, 28.2 (3C), 19.3, 17.4 (3C), 17.2, 16.9, 16.8 (3C), 16.6, 13.5, 13.0, 12.7, 12.3. HRMS (ESI) m/z: calcd for C_53_H_66_N_3_O_10_Si_2_^+^ [M + H]^+^: 960.4281; found 960.4279.

#### 3.2.5. 2′-*O*-(N-Boc-l-valinyl)-5-(perylen-3-ylethynyl)uridine **7**

To a solution of **6** (0.72 g, 0.75 mmol) in THF (20 mL), tetra-n-butylammonium fluoride trihydrate (TBAF·3H_2_O) (0.52 g, 1.65 mmol) was added, and the resulting solution was allowed to stand for 1 h at room temperature. After concentration in vacuo the residue was purified by column chromatography on silica gel (0→2% MeOH in CH_2_Cl_2_), yielding **7** (0.48 g, 90%) as a brown foam. ^1^H NMR (600 MHz, DMSO-*d_6_*): δ 11.90 (br s, 1H), 8.47 (d, J = 7.3 Hz, 1H), 8.44–8.37 (m, 2H), 8.38 (d, J = 8.0 Hz, 1H), 8.31–8.28 (m, 2H), 7.87–7.83 (m, 2H), 7.74 (d, J = 7.8 Hz, 1H), 7.69 (t, J = 7.9 Hz, 1H), 7.60–7.56 (m, 2H), 7.14 (d, J = 8.5 Hz, 1H), 6.20 (d, J = 4.8 Hz, 1H), 5.89 (d, J = 4.7 Hz, 1H), 5.29–5.25 (m, 1H), 5.21–5.16 (m, 1H), 4.19–4.14 (m, 1H), 4.01–3.96 (m, 1H), 3.92–3.88 (m, 1H), 3.73–3.66 (m, 2H), 2.02–1.95 (m, 1H), 1.38 (s, 9H), 0.81 (d, J = 6.8 Hz, 3H), 0.74 (d, J = 6.8 Hz, 3H). ^13^C NMR (150 MHz, DMSO-*d_6_*): δ 170.6, 161.2, 155.7, 148.9, 143.7, 134.1, 133.7, 131.1, 130.9, 130.5, 130.0, 129.7, 128.6, 128.3, 127.8, 127.7, 127.5, 126.9, 126.9, 125.7, 121.6, 121.3, 121.2, 120.2, 119.3, 98.1, 90.6, 88.2, 84.7, 83.4, 78.3, 77.0, 72.9, 60.1, 58.7, 29.2 (3C), 28.0, 19.0, 17.1. HRMS (ESI) m/z: calcd for C_41_H_40_N_3_O_9_^+^ [M + H]^+^: 718.2759; found 718.2763.

#### 3.2.6. 2′-*O*-l-Valinyl-5-(perylen-3-ylethynyl)uridine **8**

To a solution of **7** (0.43 g, 0.60 mmol) in a mixture of MeOH:CH_2_Cl_2_ (30 mL, 1:100, v/v), 6.6 M HCl in 1,4-dioxane (1 mL) were added, and the resulting brown solution was stirred for 12 h at room temperature. The precipitate that formed was filtered, washed with CH_2_Cl_2_ (2×10 mL), and dried in vacuo, affording **8** (0.29 g, 73%) as a brown amorphous solid. HPLC rt 7.6 min (Figure S13). ^1^H NMR (600 MHz, DMSO-*d_6_*): δ 8.89 (s, 1H), 8.53–8.47 (m, 7H), 8.47–8.44 (m, 2H), 8.20 (d, J = 8.5 Hz, 1H), 7.91 (d, J = 7.9 Hz, 1H), 7.90–7.86 (m, 2H), 7.71 (t, J = 8.0 Hz, 1H), 7.63–7.59 (m, 2H), 7.31 (s, 1H), 6.34 (d, J = 4.2 Hz, 1H), 5.52–5.49 (m, 1H), 4.31–4.28 (m, 1H), 4.10–4.07 (m, 1H), 3.82–3.78 (m, 1H), 3.75–3.69 (m, 2H), 1.91–1.84 (m, 1H), 0.74 (d, J = 7.0 Hz, 3H), 0.62 (d, J = 7.0 Hz, 3H). ^13^C NMR (150 MHz, DMSO-*d_6_*): δ 171.2, 167.6, 153.4, 153.2, 139.4, 134.0, 132.3, 131.1, 130.9, 130.1, 129.6, 128.8, 128.3, 128.3, 128.0, 127.9, 127.5, 127.0, 127.0, 124.9, 124.4, 121.8, 121.5, 121.3, 120.3, 107.1, 104.0, 86.3, 86.0, 77.6, 73.6, 60.4, 57.3, 28.7, 17.5, 17.3. HRMS (ESI) m/z: calcd for C_36_H_32_N_3_O_7_^+^ [M-Cl^-^]^+^: 618.2235; found 618.2225.

#### 3.2.7. 3-Methyl-2-(2-(perylen-3-yl)vinyl)benzo[d]thiazol-3-ium chloride **10**

A solution of perylene-3-carbaldehyde (0.28 g, 1.0 mmol) and 2,3-dimethylbenzo[*d*]thiazol-3-ium chloride **9** (0.24 g, 1.2 mmol) in Ac_2_O (5 mL) was heated at 150 °C for 2 h. Then, water (2 mL) was added, and the resulting dark purple solution was heated at 50 °C for 30 min. After concentration in vacuo, the residue was purified by column chromatography on silica gel (0→12% MeOH in CH_2_Cl_2_), yielding **10** (0.27 g, 54%) as a dark purple amorphous solid. HPLC rt 9 min (Figure S14). ^1^H NMR (600 MHz, DMSO-*d_6_*): δ 8.75 (d, J = 15.5 Hz, 1H), 8.56 (d, J = 7.4 Hz, 1H), 8.52 (d, J = 7.9 Hz, 2H), 8.49–8.42 (m, 3H), 8.36 (d, J = 8.4 Hz, 1H), 8.26 (d, J = 8.4 Hz, 1H), 8.16 (d, J = 15.5 Hz, 1H), 7.92–7.83 (m, 3H), 7.81–7.73 (m, 2H), 7.62 (t, J = 7.8 Hz, 1H), 7.58 (t, J = 7.7 Hz, 1H), 4.40 (s, 3H). ^13^C NMR (150 MHz, DMSO-*d_6_*): δ 171.2, 143.5, 141.9, 134.8, 133.9, 132.5, 131.0, 129.9, 129.8, 129.5, 129.5, 129.3, 128.5, 128.3 (2C), 128.2, 128.1, 127.9, 127.3, 127.1, 127.0, 124.0, 123.1, 122.7, 121.8, 121.4, 120.5, 116.7, 115.1, 36.3. HRMS (ESI) m/z: calcd for C_30_H_20_NS+ [M-Cl^-^]^+^: 426.1311; found 426.1299.

### 3.3. Solubility

The samples were dissolved in DMSO, diluted in series, and photometrically analyzed. The average molar absorption coefficient was calculated from the obtained optical densities of calibration solutions at the absorption maxima. Solubility values were determined as previously described [52] by adding weighed amounts of compounds to distilled water followed by sonication (30 min), centrifugation (12,000 rpm, 10 min), and photometry.

### 3.4. In Vitro Biological Studies

#### 3.4.1. Cells and Viruses

The green monkey kidney (Vero) cell line originated from WHO Biologicals, Switzerland (10–87), and the Madin-Darby canine kidney (MDCK) cell line was obtained from the American Type Culture Collection (ATCC CCL-34).

Viruses and strains used in the present work were severe acute respiratory syndrome-related coronavirus 2 (SARS-CoV-2, strain PIK35, GISAID EPI_ISL_428852), Chikungunya virus (CHIKV, strain Nic, GenBank IDs MN271691-2) and influenza A virus (H1N1, strain A/PR/8/34, ATCC VR-95).

#### 3.4.2. Methods

##### Cell viability Assay in Vero Cells

Two-fold dilutions of the compounds (starting from 100 μM) were prepared in DMEM (Chumakov FSC R&D IBP RAS, Russia) and added to confluent cell monolayers. After 5 days of incubation (37 °C, 5% CO_2_), the cultural supernatant was substituted with a resazurin solution (25 μg/mL), followed by 4 h incubation (37 °C, 5% CO_2_). Then, 20 µL of 10% SDS were added to stop the reaction. Fluorescence intensity was measured with Promega GloMax-Multi Detection System (Ex525/Em580–640). Cells treated with dilutions of the compounds and DMSO, but not resazurin, were used to subtract the background fluorescence. All experimental procedures were performed in two replicates. Fluorescence curves were analyzed with Microsoft Excel 2013. 50% Cytotoxic concentrations (CC_50_) were calculated.

##### Cell Viability Assay in MDCK Cells

Compound solutions were prepared in DMSO (20 mg/mL), and then 2-fold dilutions in cultural medium were prepared starting from 1000 μg/mL. One-day MDCK cell monolayers (over 95% confluency) in 96-well plates (6×105 cells/well) were washed twice with serum-free alpha-MEM (Thermo-Fisher Scientific, USA) and treated with 100 μL of compound dilutions in 2 replicates. The plates were then incubated for 24 h at 37 °C in a CO_2_ incubator. Cell viability was assessed using the tetrazolium test (MTT, ApplyChem) [53]. Optical density was measured on a Wallac 1420 Victor2 microplate reader (Perkin Elmer, Ramsey, MN, USA) at 535 nm, then CC_50_ values were calculated.

##### IAV Virus Yield Reduction Assay

Compound solutions were prepared in DMSO (20 mg/mL). The compounds were weighed in 2 mg amounts and dissolved in 100 μL of DMSO. Then, a series of 3-fold dilutions was prepared, starting from 300 µg/mL, in a cultural medium. The MDCK cell monolayer was treated with compound dilutions and incubated for 1 h in a CO_2_ incubator at 37 °C. Untreated cells were used as negative control. Afterward, the virus was added (MOI 1), and cells were incubated for 24 h at 37 °C in a CO_2_ incubator. After incubation, the culture medium was collected, and viral yields were determined by titration. In brief, a series of 10-fold dilutions (10^−7^–10^−1^) of viral supernatants were prepared in a cultural medium and applied to daily MDCK cell monolayers. Infected cells were incubated for 72 h at 37 °C in a CO_2_ incubator. The presence of the virus in the culture supernatants was determined using the hemagglutination assay with a 1% suspension of chicken erythrocytes [54]. Viral titer was calculated according to the Reed-and-Muench method and was expressed as 50% tissue infectious doses (TID_50_) per 100 μL of volume. Inhibition (%) was calculated by comparison with non-treated cells, and EC_50_ was calculated from inhibitory curves.

##### SARS-CoV-2 and CHIKV Cytopathic Effect (CPE) Inhibition Test

The procedure was described previously [55]. In brief, two-fold dilutions of the compounds were prepared in Dulbecco MEM (Chumakov FSC R&D IBP RAS, Russia) and mixed 1:1 with a virus suspension containing 50–200 TCID_50_ and incubated for 1 h at 37 °C in a CO_2_ incubator. The final concentration series started from 100 μM. Then, compound-virus mixtures were added to confluent Vero cell monolayers in 2 replicates. After incubation at 37 °C in a CO_2_-incubator for 5 days, CPE signs (cell death) were assessed via microscope, and 50% effective concentration (EC_50_) (compound concentration required to decrease CPE by 50%) was calculated using the Karber method [56].

##### Plasma Stability Test

In vitro stability of 10 was studied in Gibco FBS (Thermo-Fisher Scientific, USA) by following the reported procedure with minor modifications [57]. A 10 mM solution of the compound in DMSO (20 μL) was added to 80% FBS in 0.05 M PBS (pH 7.4) at 37 °C to yield the final concentration of 200 μM. The assays were carried out in triplicate in a water bath at 37 °C. Samples (100 μL) were taken at 0, 15, 30, 45, 60, 90, 120, 180, 240, and 300 min and added to 400 μL acetonitrile in order to deproteinize the plasma. The samples were then shaken for 1 min and centrifuged at 37 °C at 15,000 rpm for 5 min. The supernatants were analyzed by HPLC (detection at 530 nm). AUC values were determined and averaged from three independent experiments. In vitro serum half-life (t_1/2_) was calculated using the equation t_1/2_ = 0.693/b, where b is the slope found by linear fitting of the negative natural logarithm of the fraction remaining (AUC_0-300_/AUC_0_) vs. incubation time.

### 3.5. In Vivo Biological Studies

#### 3.5.1. Animals

Female BALB/c mice (5–7 weeks old) were obtained from the “Stolbovaya” branch of the Scientific center for biomedical technology of the Federal medical-biological agency (Moscow region, Russia). All animal procedures were performed in accordance with the Helsinki declaration and Russian animal protection regulations.

#### 3.5.2. Virus

Influenza A virus strain A/California/7/09/MA adapted to mice by 8 consecutive passages and accumulated on chicken embryos was used.

#### 3.5.3. Design of the Experiment to Determine the Antiviral Activity of Compounds

The test compound (0.5 and 4.5 mg/kg), placebo (PBS as a negative control), and reference drug Tamiflu (20 mg/kg, oseltamivir phosphate as a positive control) were administered once per day to the animals using a gastric tube in a 0.2 mL volume according to the therapeutic and prophylactic scheme (24 and 1 h before infection and 1, 2, and 3 days post infection). The mice were intranasally infected with 5 MLD_50_ of virus in a 50 μL volume. The animals were observed and weighed daily for 14 days post infection. Kaplan–Meier survival curves were built with GraphPad Prism 6.0. The Mantel–Cox test was used for posterior pairwise comparisons with the Placebo group. The protection index was calculated as a ratio (Mc − Me)/Mc × 100%, where Mc and Me are mortality rates in the placebo and experimental groups, respectively, at the end of the experiment (14 days post infection). Differences in weight between the groups were established with one-way ANOVA with Dunnett’s test for pairwise comparisons.

## 4. Conclusions

While previously reported data on perylene RAFIs have demonstrated remarkable subnanomolar antiviral activity in vitro, low water solubility and a rather controversial mode of action have discouraged in vivo studies. The introduction of positively charged moieties led to a dramatic improvement in the solubility of these compounds without a significant reduction in efficacy in vitro. We demonstrated 22% protective activity and accelerated weight recovery upon intragastrical administration of the most promising compound **10** in a mouse model of lethal influenza pneumonia, but the differences were not significant. This is the first example of in vivo efficacy assessment of RAFIs, paving the way for the development of water-soluble perylene-based antiviral agents and their preclinical studies.

## Figures and Tables

**Table 1:** In vitro antiviral activity and cytotoxicity of perylene-based compounds against CHIKV, SARS-CoV-2, and IAV (M ± SD).

Compound	Antiviral Activity, EC_50_ ± SD, µM			Cytotoxicity, CC_50_ ± SD, µM	
	CHIKV	SARS-CoV-2	IAV	Vero	MDCK
				5 d	24 h
**2a**	0.47 ± 0.16	11.3 ± 1.8	20.6 ± 8	>100	>100
**2b**	>100	>100	62 ± 22	>100	>100
**3a**	11.3 ± 9.0	1.88 ± 0.88	49 ± 19	>100	>100
**3b**	10.7 ± 2.6	9.2 ± 4.3	>100	>100	>100
**5**	1.17 ± 0.55	>100	ND	>100	ND
**6**	>100	>100	31 ± 10	>100	>100
**7**	<0.016	0.075 ± 0.018	4.6 ± 1.0	98 ± 17	62 ± 24
**8**	0.55 ± 0.33	1.28 ± 0.40	3.8 ± 1.3	>100	>100
**10**	0.99 ± 0.15	1.45 ± 0.95	22.7 ± 10.0	>100	69 ± 27
Positive control	NHC ^a^ 9.7 ± 2.8	NHC ^a^ 7.4 ± 3.6	Tamiflu ^b^ 0.02 ± 0.01	NHC ^a^ >100	Tamiflu ^b^ >100

^a^ N^4^-hydroxycytidine; ^b^ Oseltamivir phosphate.

**Table 2:** Solubility of perylene-based compounds in water.

Compound	ε, M^−1^·cm^−1^	Solubility in Water, mM
**3a**	20,000	3.4
**3b**	20,000	5.0
**8**	34,000	0.1
**10**	60,000	11.0
**aUY11** [37]	41,000	insoluble

**Table 3:** Infection characteristics of BALB/c mice with experimental influenza pneumonia upon infection with influenza A/California/7/09/MA virus.

Cmpd	Dose, mg/kg	Mortality Rate, %	*p* (Mortality Rates Comparison)	Average Life Expectancy (M ± SEM), days	*p* (Average Life Expectancy Comparison)	Protective Index (PI)
Placebo	–	90	–	8.40 ± 0.9	–	0
Tamiflu	20	40	0.02084 *	11.4 ± 1.1	0.0134 *	56
**10**	4.5	70	0.7295	8.6 ± 1.2	0.4483	22
**10**	0.5	80	0.7362	8.6 ± 1.0	0.4524	11

* Difference between Placebo and Tamiflu groups is statistically significant (*p* values < 0.05 are shown in bold).

## Data Availability

Not applicable.

## Supplementary Materials

The following supporting information can be downloaded at: https://www.mdpi.com/article/10.3390/ph15101178/s1, Figures S1–S14: ^1^H and ^13^C NMR spectra of the compounds, Plasma stability evaluation, HPLC trace for compounds; Table S1: Predicted ADMET profiles of the synthesized compounds.
