# Supplementary material for: Cationic Perylene Antivirals with Aqueous Solubility for Studies In Vivo
Source: Pharmaceuticals (Basel). 2022 Sep 22;15(10):1178. doi: 10.3390/ph15101178 (PMC9610897; doi:10.3390/ph15101178)
Supplement: Supplementary file 1 [file pharmaceuticals-15-01178-s001.zip › pharmaceuticals-1916112-supplementary.pdf]

Cationic Perylene Antivirals with Aqueous Solubility for Studies in vivo

Supplementary materials

Anna A. Shtro [1,†,*], Anzhelika V. Garshinina [1,†], Vera A. Alferova [2,3,†], Polina N. Kamzeeva [2], Viktor P. Volok [2,4], Ekaterina S. Kolpakova [4], Timofei D. Nikitin 2, Alexey A. Chistov 2, Evgeny S. Belyaev [5], Vladimir A. Korshun [2], Liubov I. Kozlovskaya [4,6,*] and Andrey V. Aralov[2,*]

[1] Smorodintsev Research Institute of Influenza, Saint Petersburg, 197376, Russia
[2] Shemyakin-Ovchinnikov Institute of Bioorganic Chemistry, Russian Academy of Sciences, Moscow, 117997, Russia
[3] Gause Institute of New Antibiotics, Moscow, 119021, Russia
[4] Chumakov Scientific Center for Research and Development of Immune-and-Biological Products (Institute of poliomyelitis), Russian Academy of Sciences, Moscow, 108819, Russia
[5] Frumkin Institute of Physical Chemistry and Electrochemistry, Russian Academy of Science, Moscow, 119071, Russia
[6] Institute of Translational Medicine and Biotechnology, Sechenov Moscow State Medical University, Moscow, 119991, Russia
* Correspondence: aashtro@gmail.com (A.A.S.); lubov_i_k@mail.ru (L.I.K.); baruh238@mail.ru (AVA)
† These authors contributed equally to this work.

CONTENTS

Figure S1. ^{1}H and ^{13}C NMR spectra of the compound **2a**. 3
Figure S2. ^{1}H and ^{13}C NMR spectra of the compound **2b**. 4
Figure S3. ^{1}H and ^{13}C NMR spectra of the compound **3a**. 5
Figure S4. ^{1}H and ^{13}C NMR spectra of the compound **3b**. 6
Figure S5. ^{1}H and ^{13}C NMR spectra of the compound **5**. 7
Figure S6. ^{1}H and ^{13}C NMR spectra of the compound **6**. 8
Figure S7. ^{1}H and ^{13}C NMR spectra of the compound **7**. 9
Figure S8. ^{1}H and ^{13}C NMR spectra of the compound **8**. 10
Figure S9. ^{1}H and ^{13}C NMR spectra of the compound **10**. 11
Table S1. Predicted ADMET profiles of the synthesized compounds.......... 12
Figure S10. Serum stability evaluation 14
Figure S11. HPLC trace for compound **3a** 15
Figure S12. HPLC trace for compound **3b** 16
Figure S13. HPLC trace for compound **8** 17
Figure S14. HPLC trace for compound **10** 18

Figure S1. ^{1}H and ^{13}C NMR spectra of the compound **2a**.

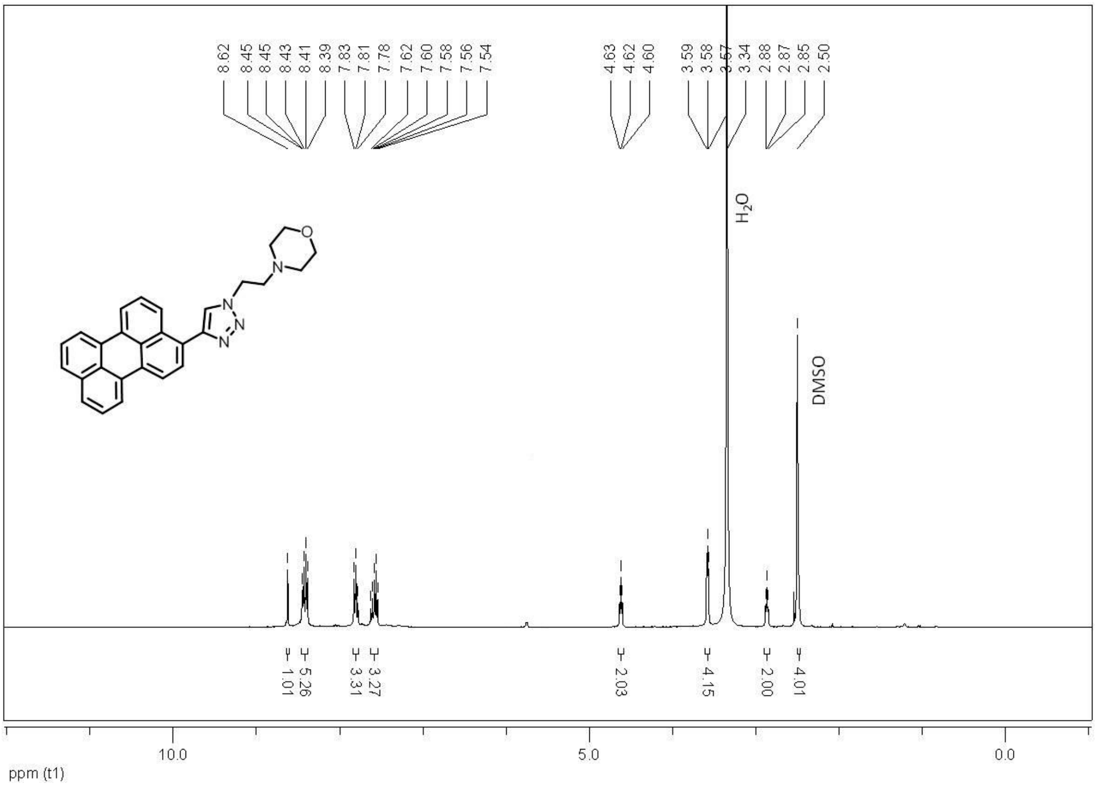

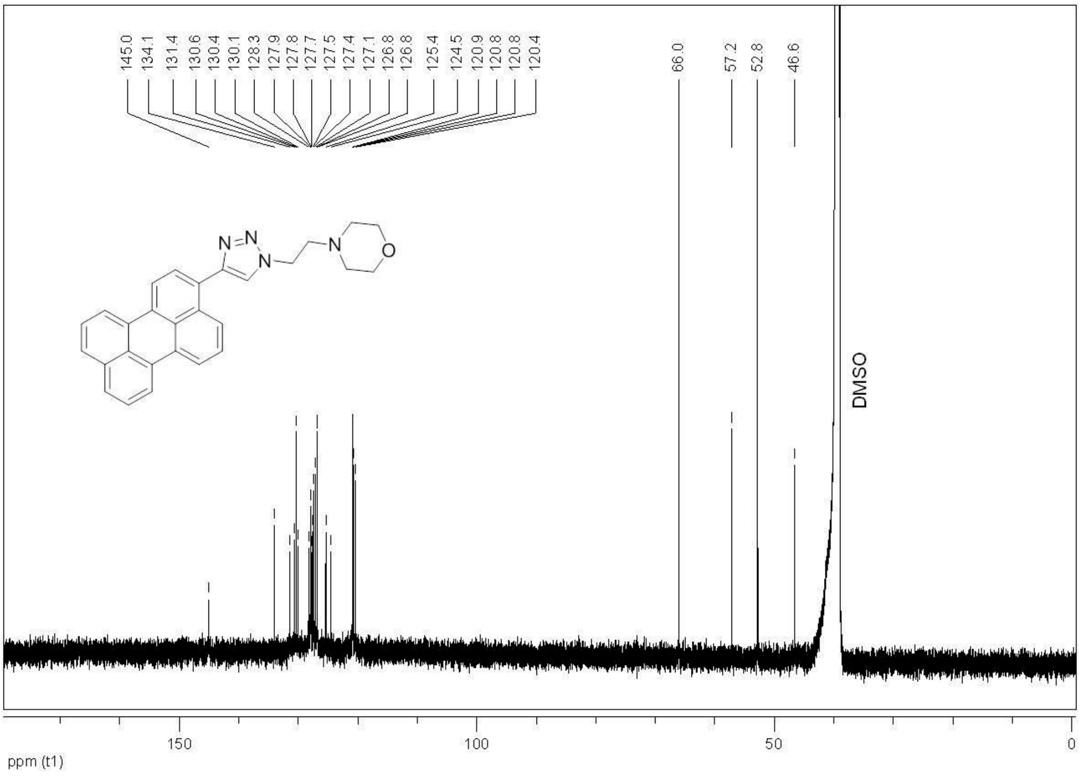

Back to Contents

Figure S2. ^{1}H and ^{13}C NMR spectra of the compound **2b**.

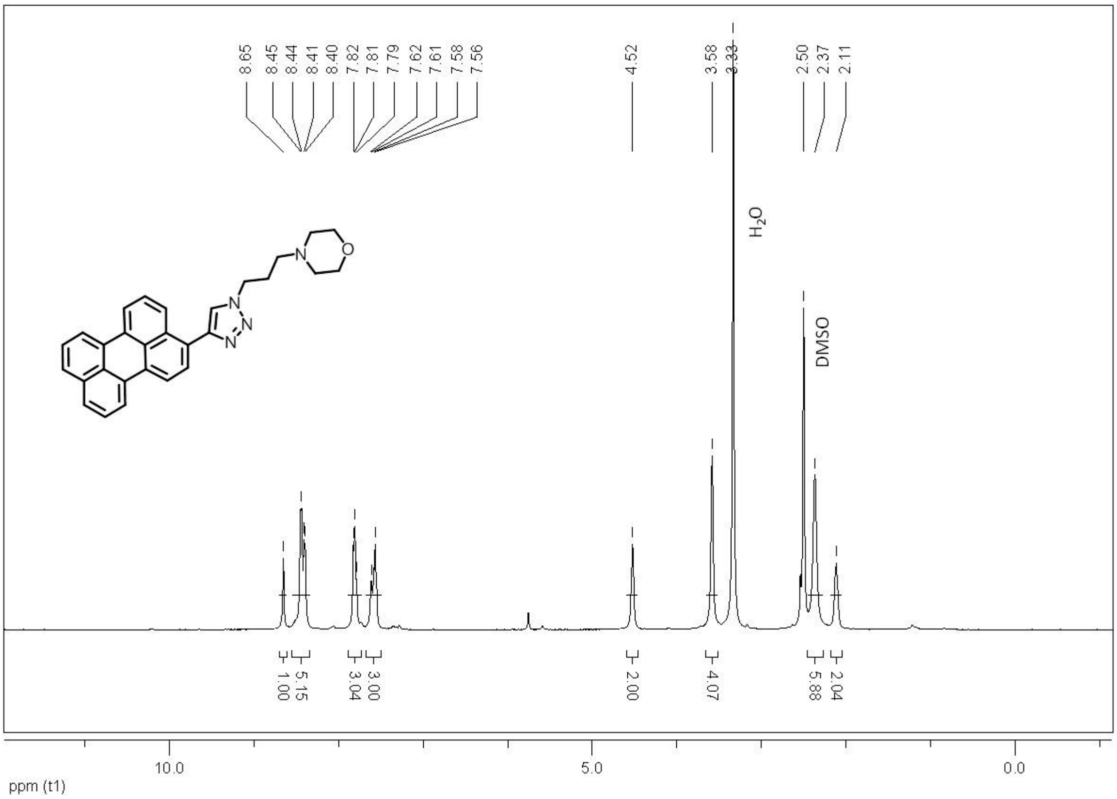

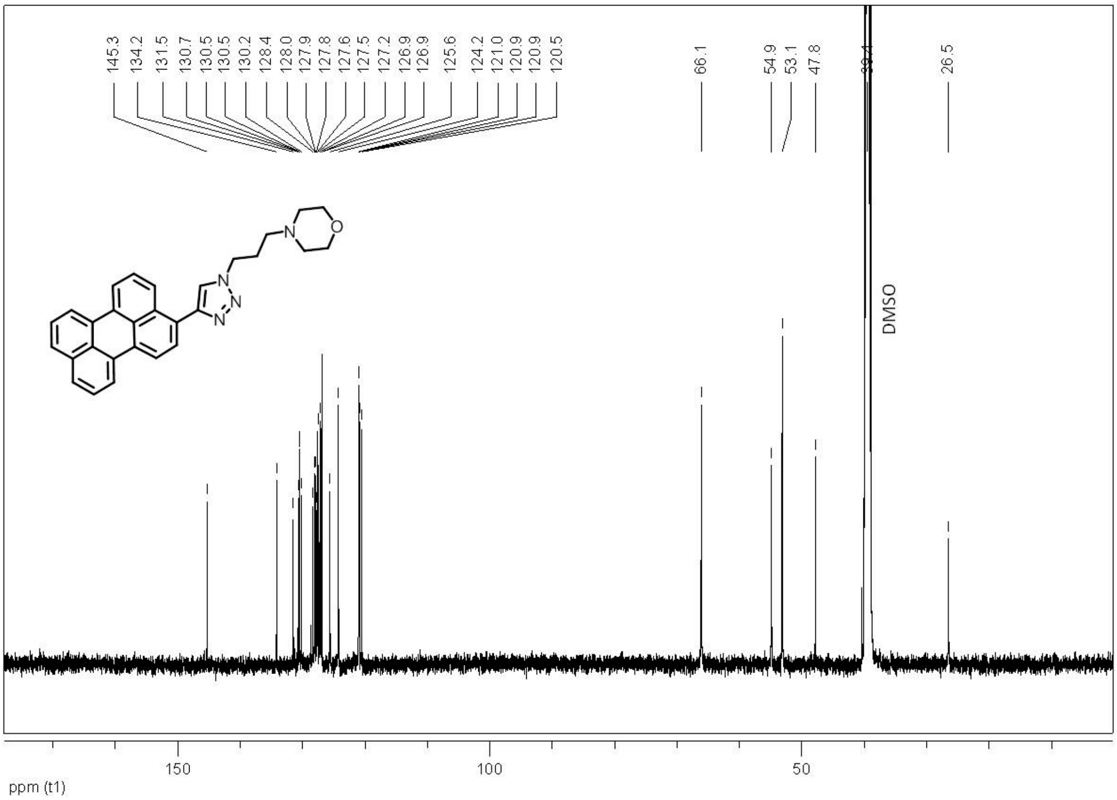

Back to Contents

Figure S3. ^{1}H and ^{13}C NMR spectra of the compound **3a**.

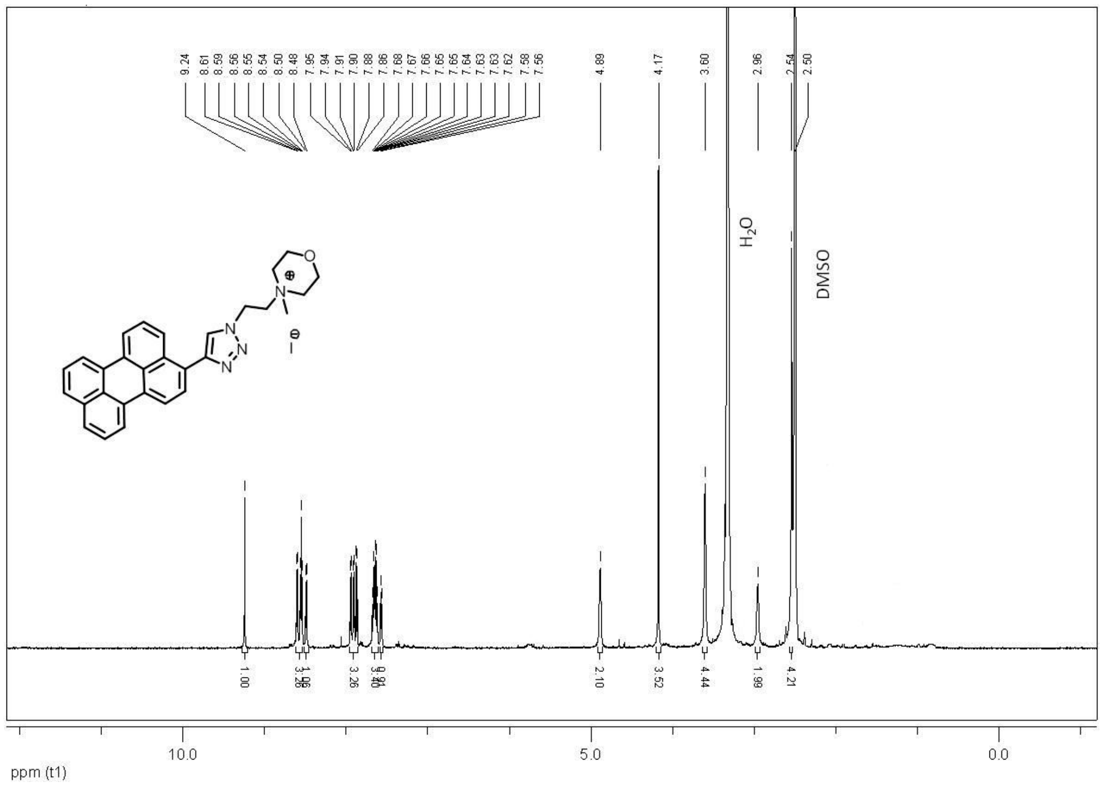

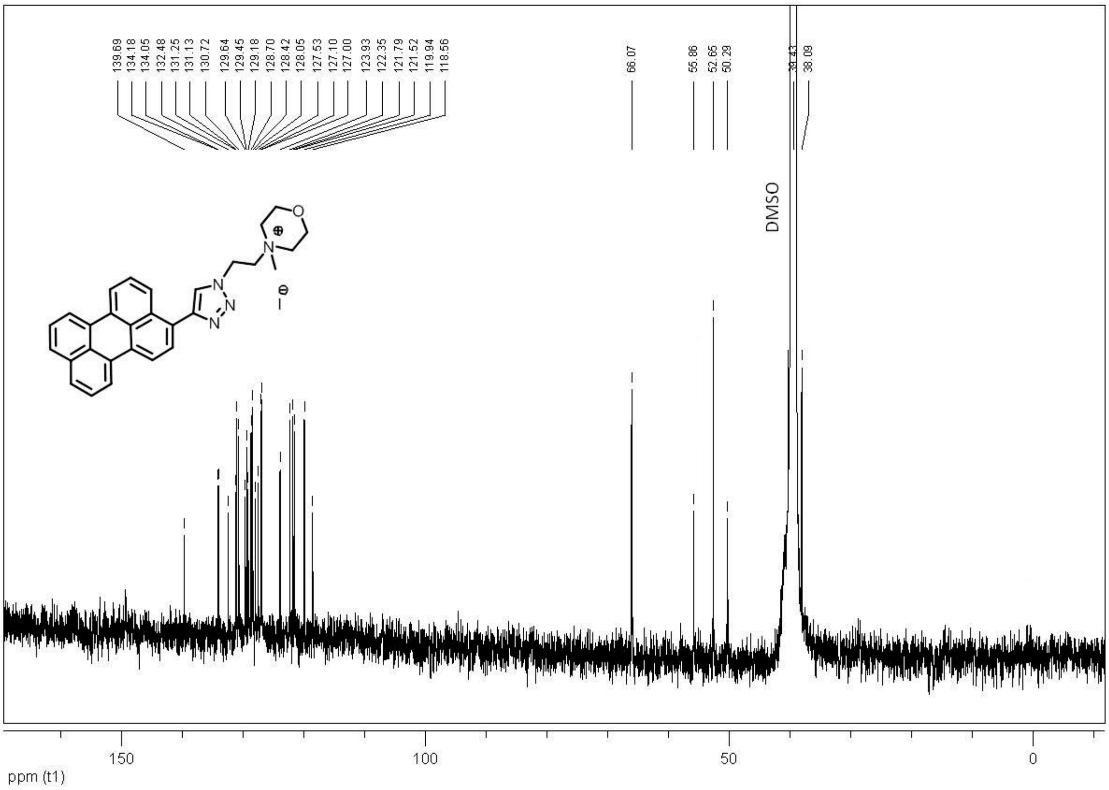

Back to Contents

Figure S4. ^{1}H and ^{13}C NMR spectra of the compound **3b**.

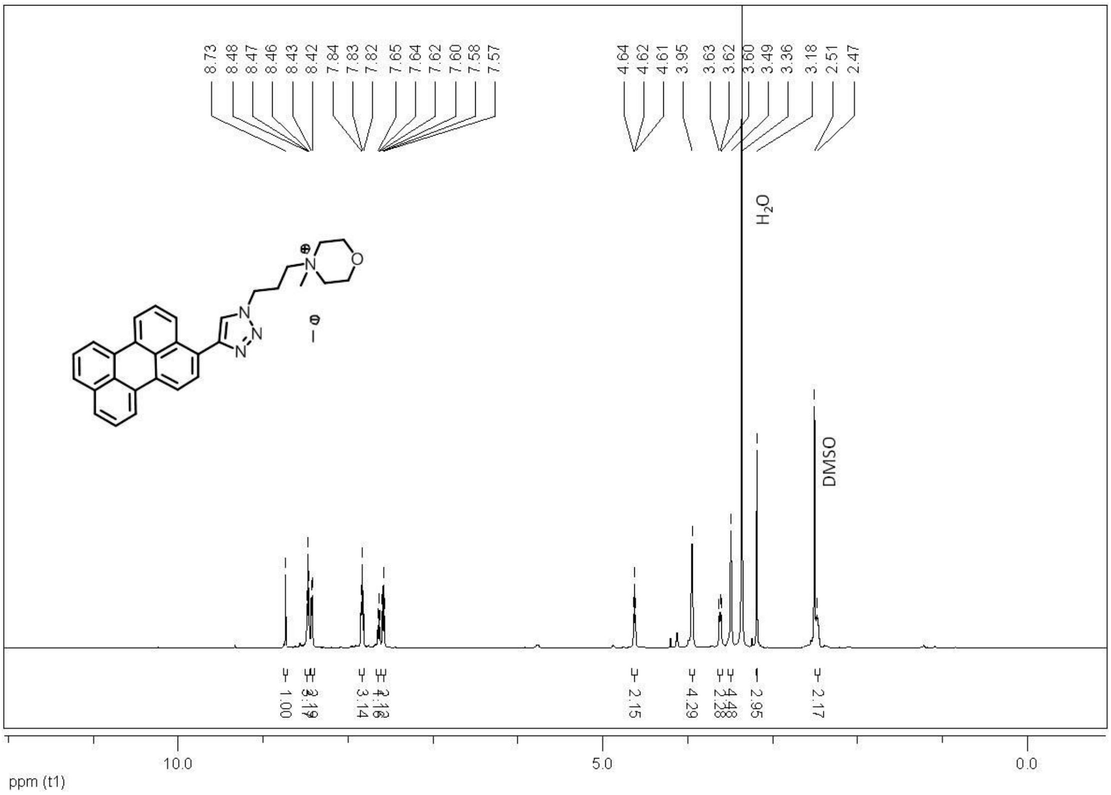

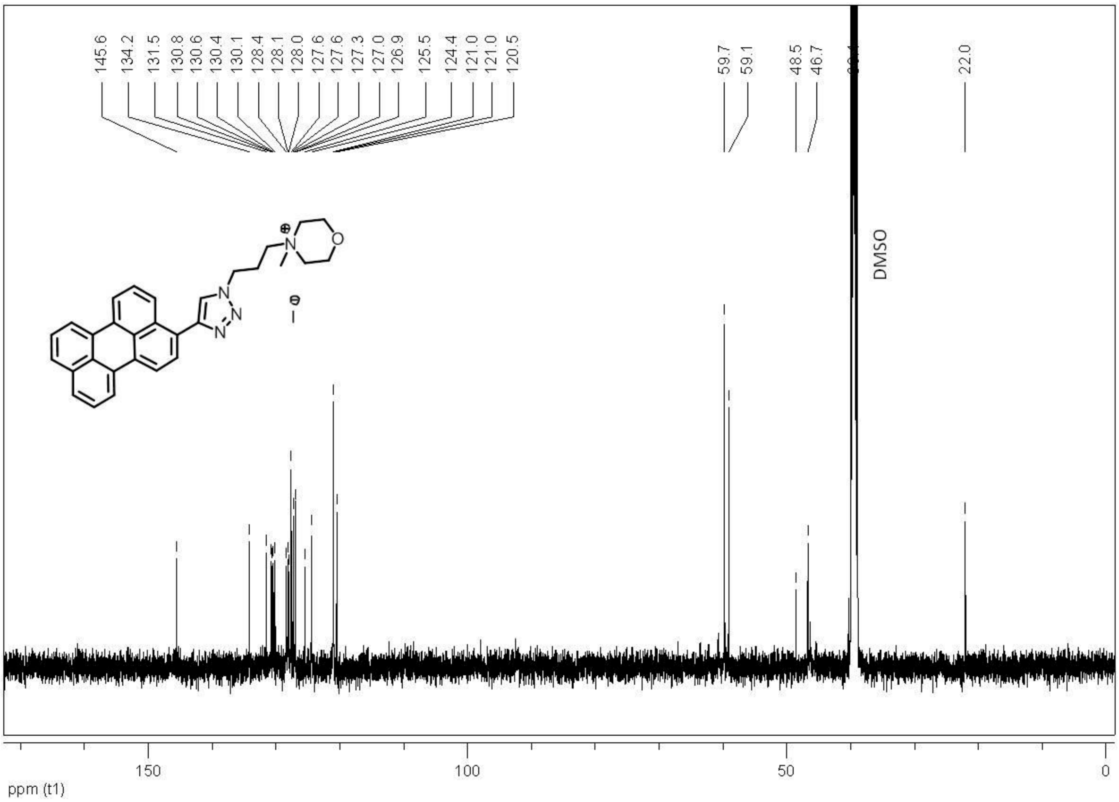

Back to Contents

Figure S5. ^{1}H and ^{13}C NMR spectra of the compound **5**.

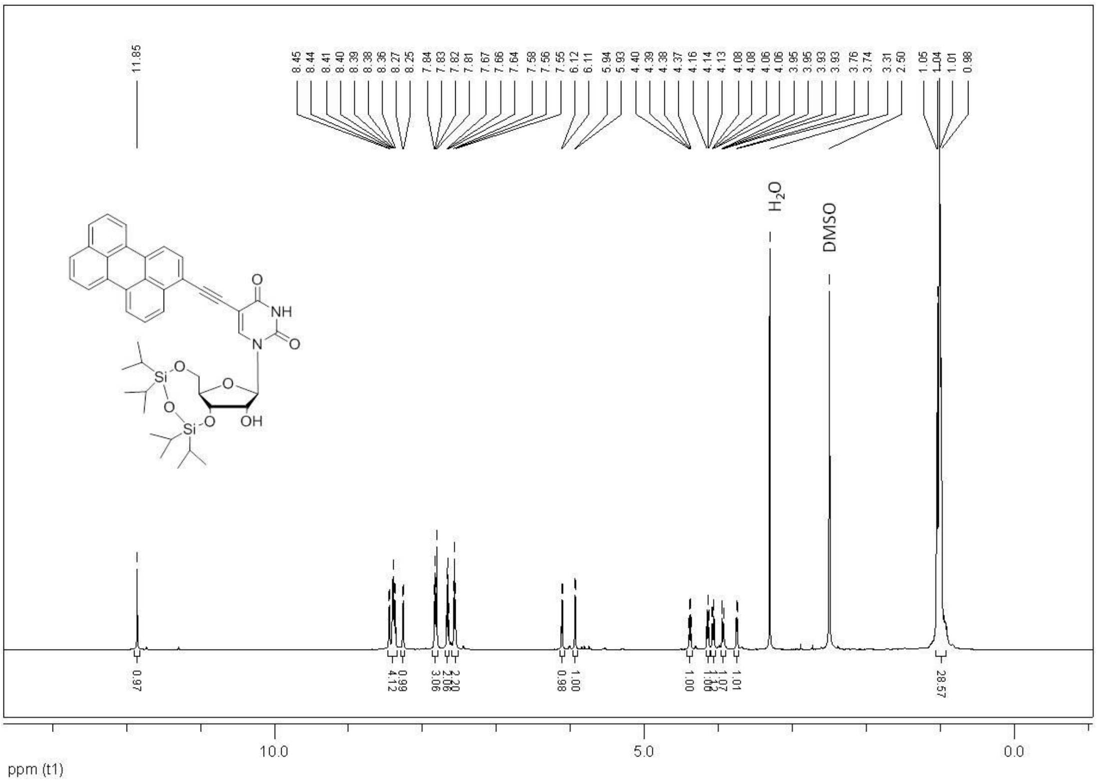

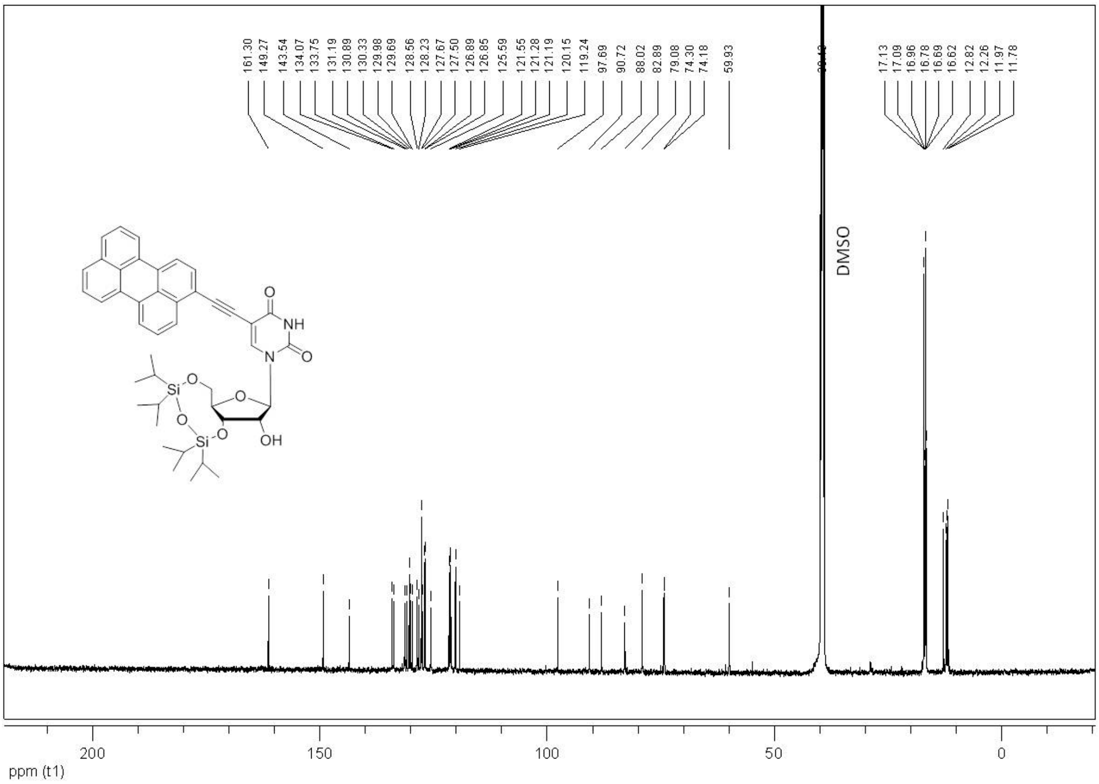

Back to Contents

Figure S6. ^{1}H and ^{13}C NMR spectra of the compound **6**.

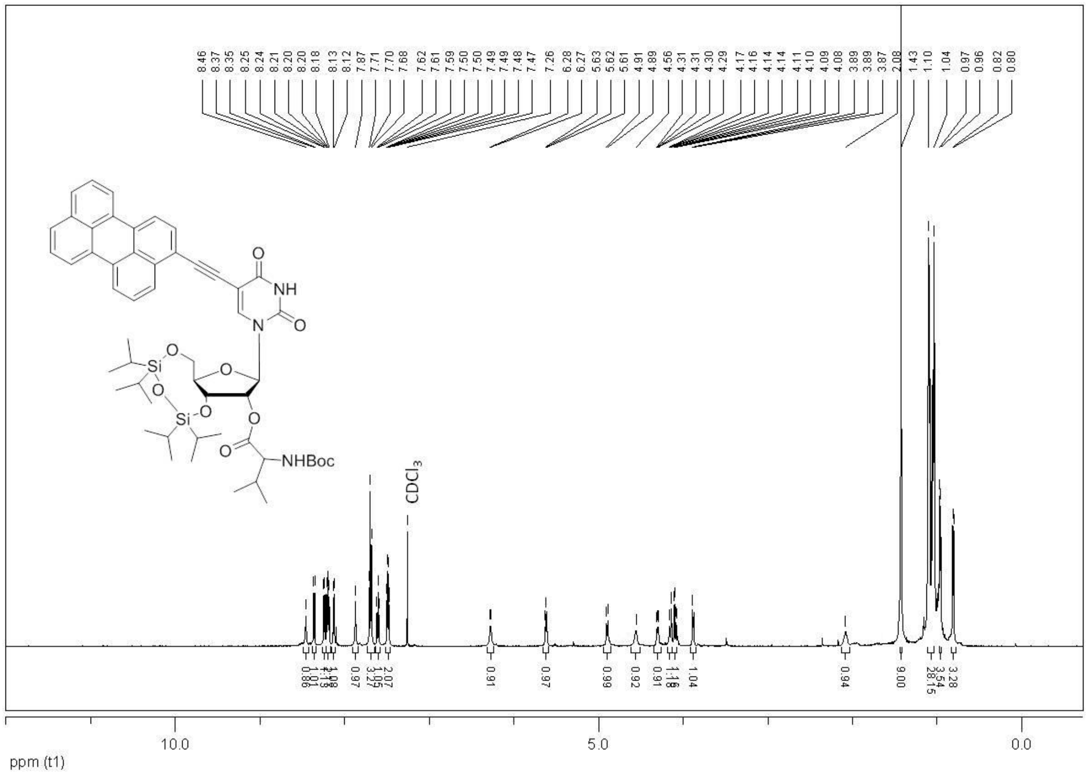

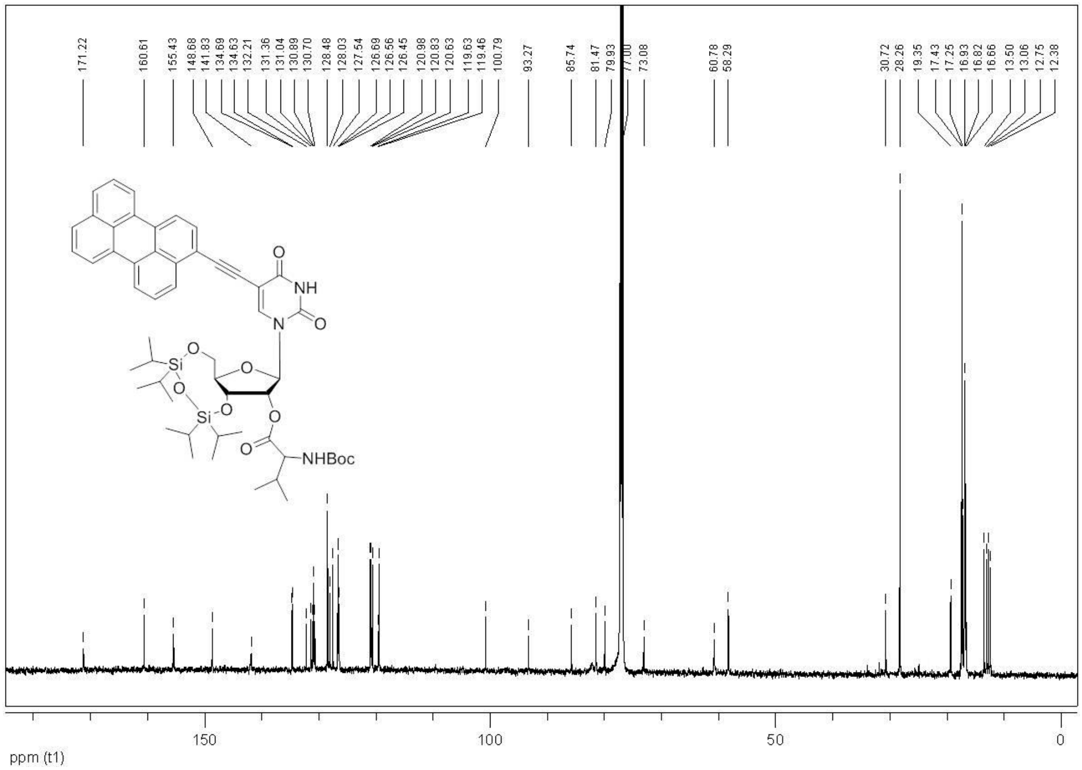

Back to Contents

Figure S7. ^{1}H and ^{13}C NMR spectra of the compound **7**.

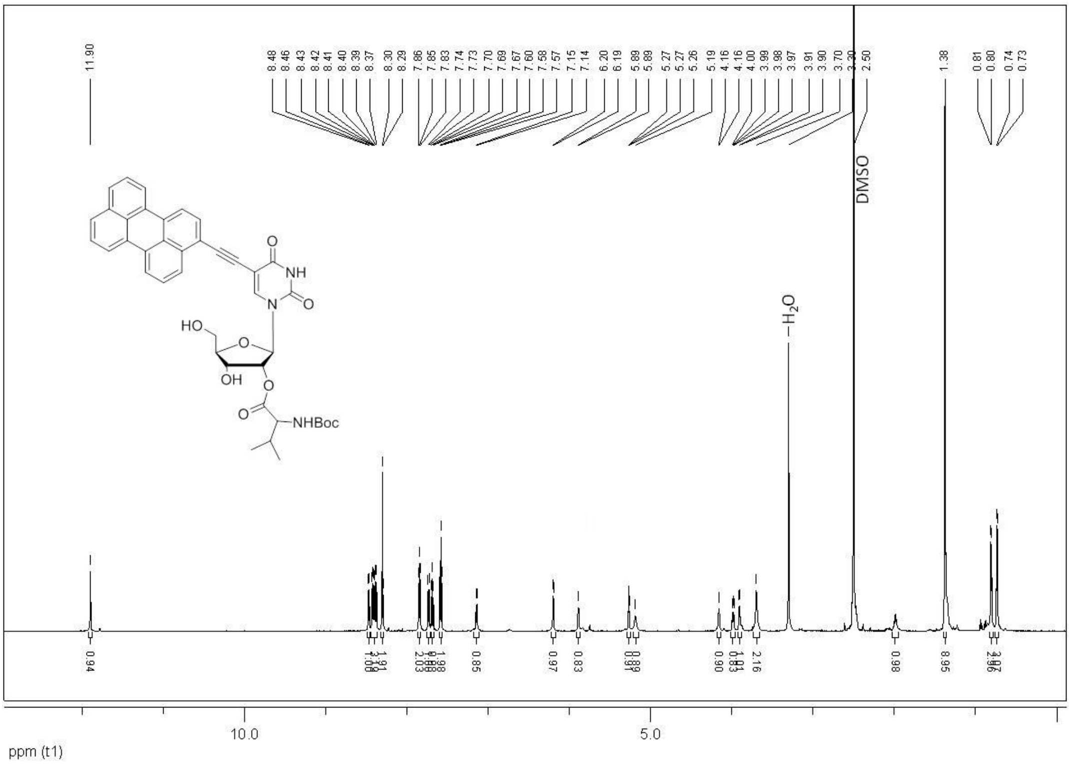

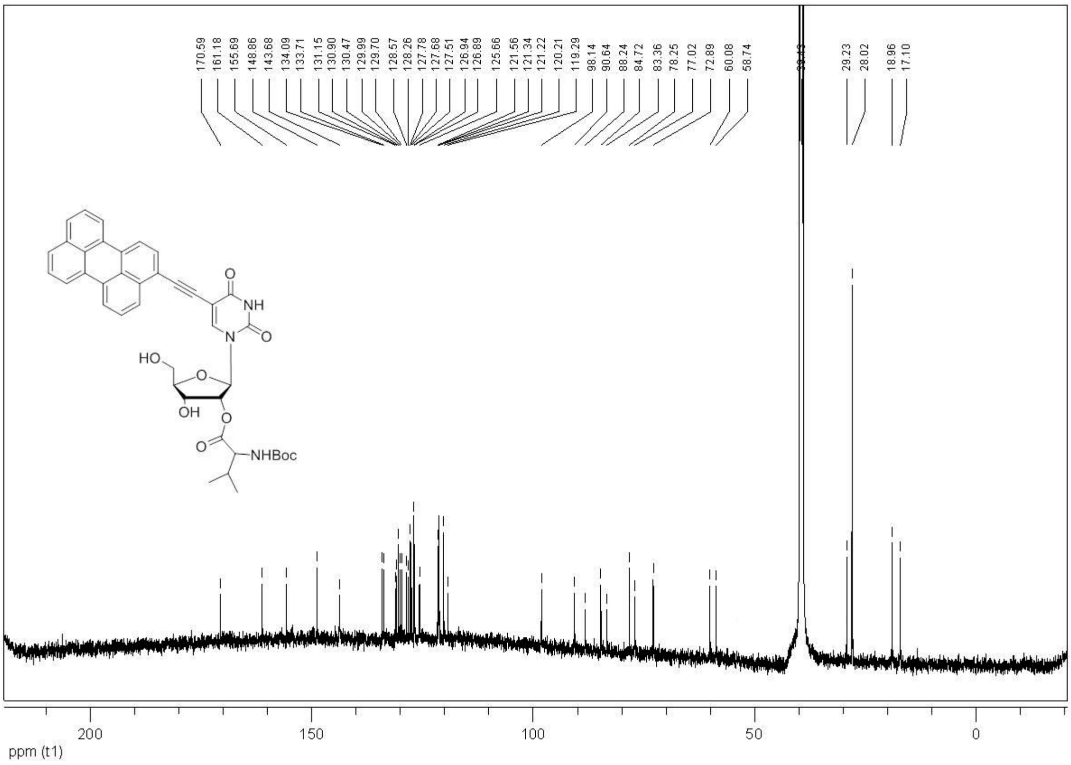

Back to Contents

Figure S8. ^{1}H and ^{13}C NMR spectra of the compound **8**.

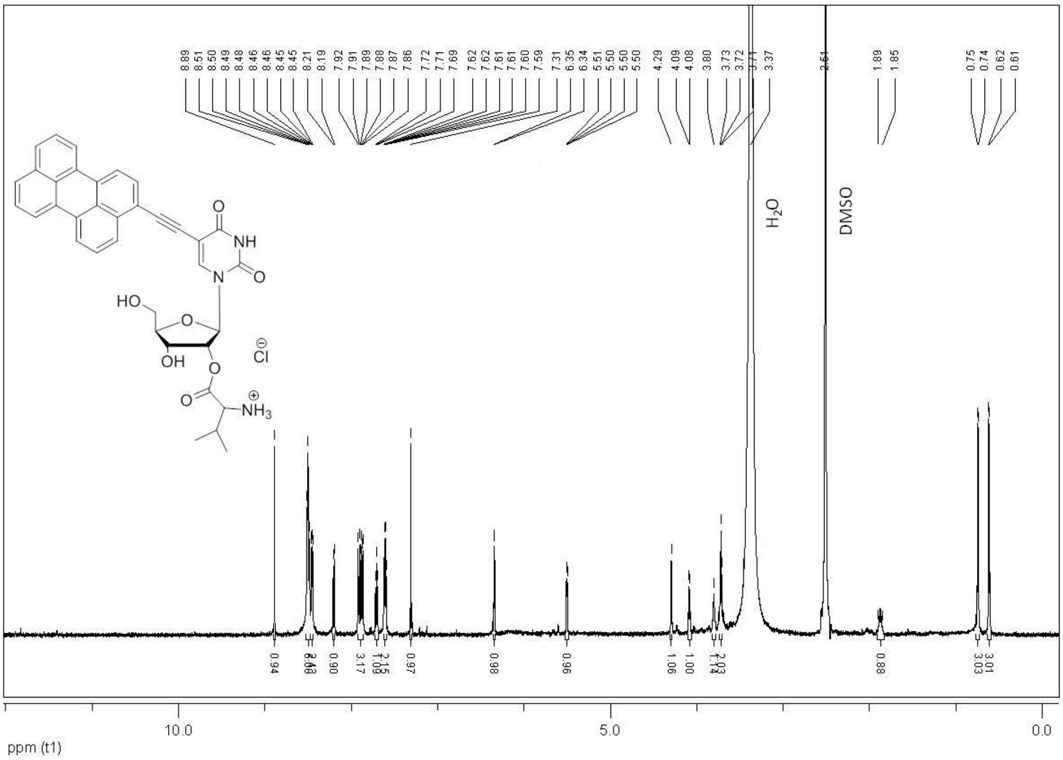

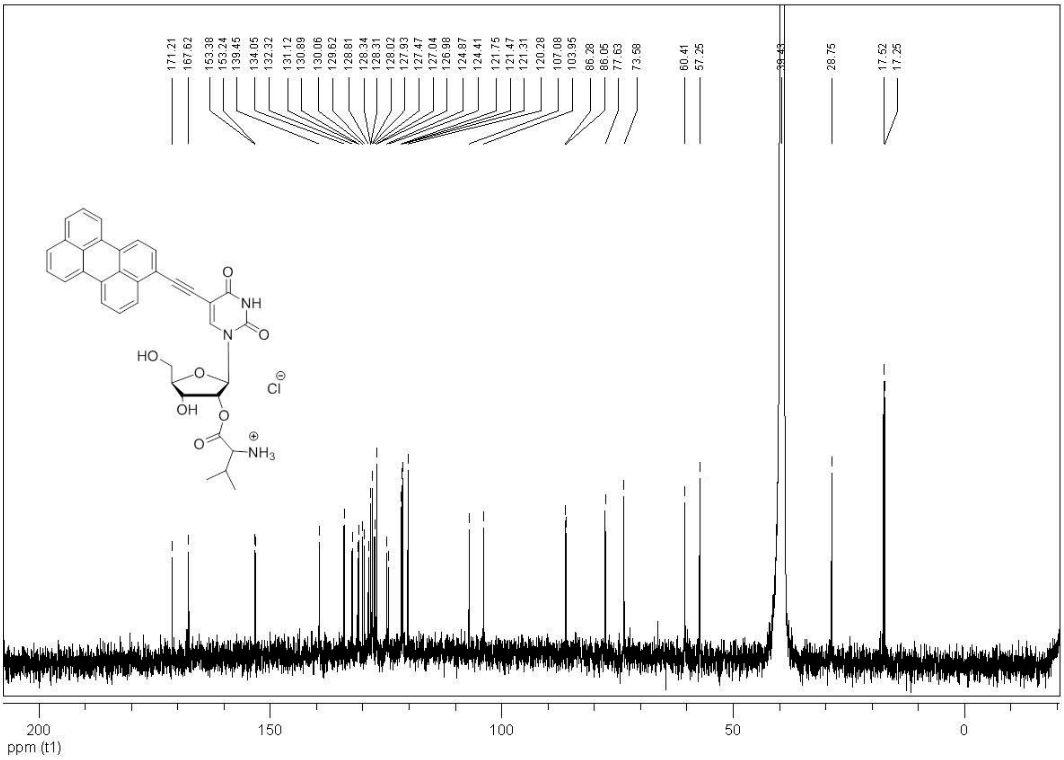

Back to Contents

Figure S9. ^{1}H and ^{13}C NMR spectra of the compound **10**.

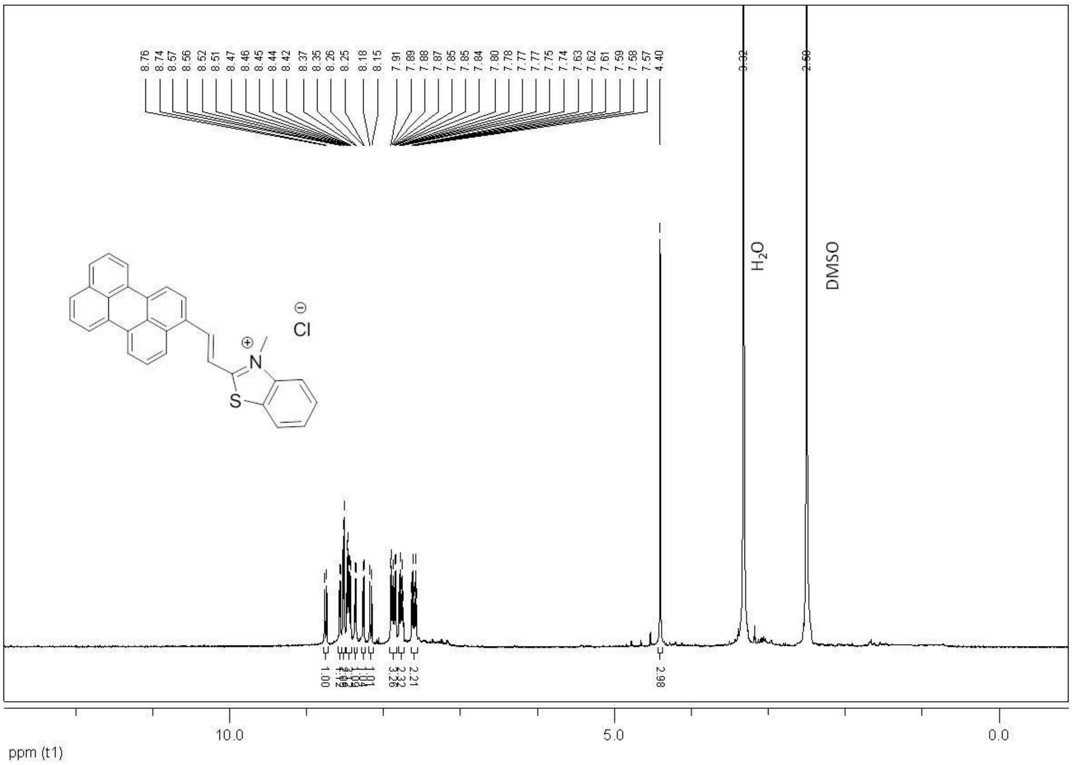

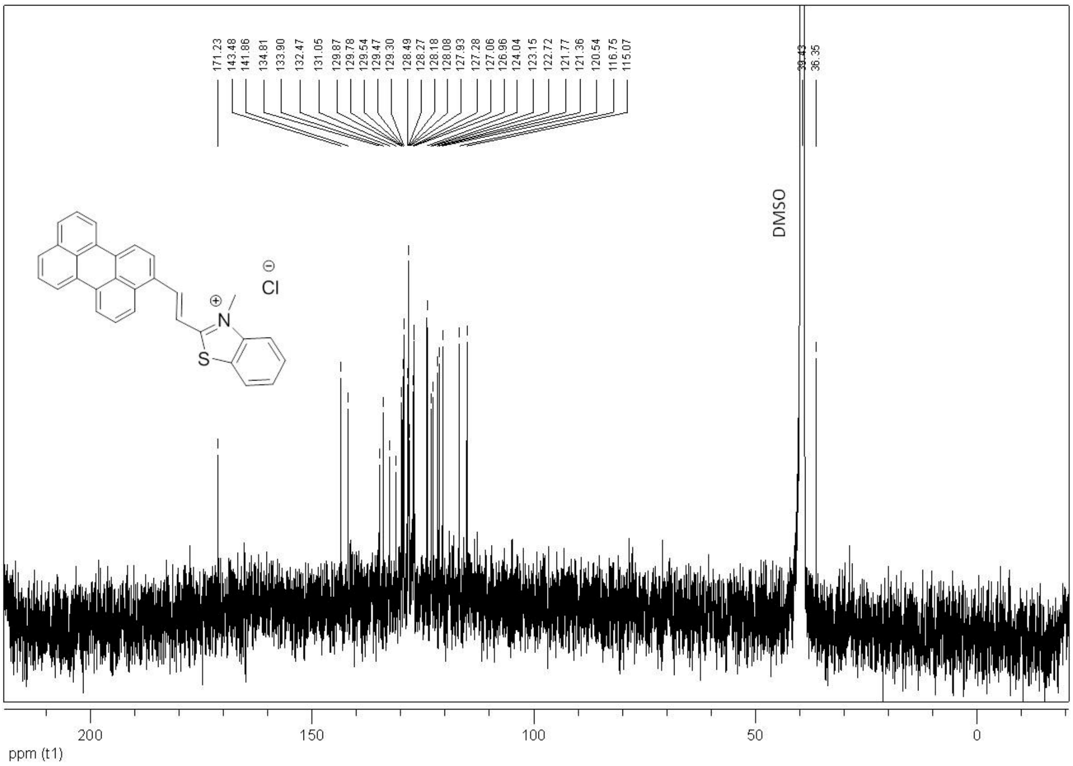

Back to Contents

Table S1. Predicted ADMET profiles of the synthesized compounds

compound	Structure	cHIA[a]	clogBB[b]
**2a**	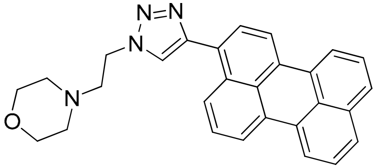	85.4	0.17
**2b**	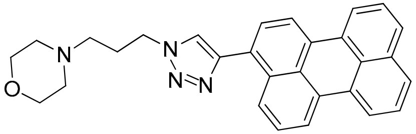	99.97	0.25
**3a**	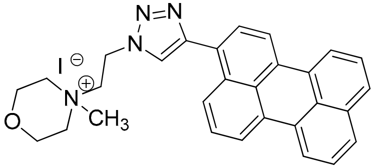	10.06	0.04
**3b**	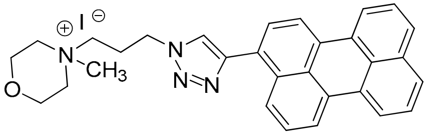	10.06	0.13
**5**	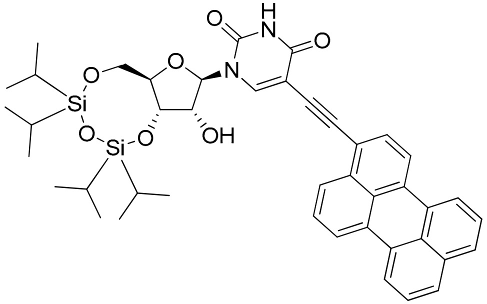	83.25	-0.67

Back to Contents

**6**	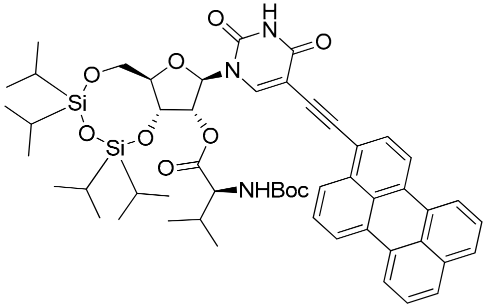	73.26	0.03
**7**	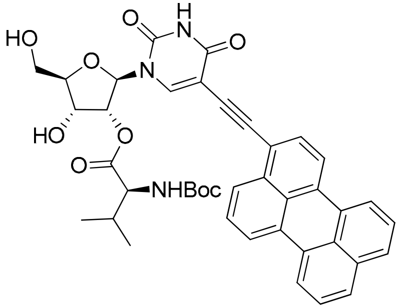	42.53	0.53
**8**	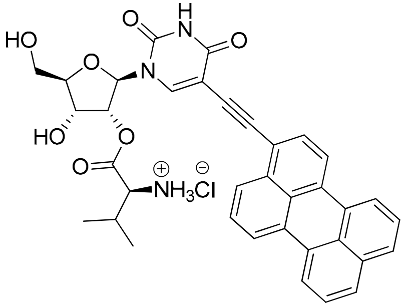	42.53	-0.6
**10**	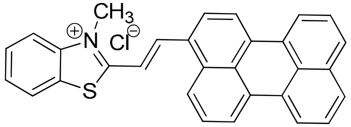	97.22	0.01

[a] HIA – human intestinal absorption [%],

[b] LogBB – blood-brain barrier permeability.

Figure S10. Serum stability evaluation

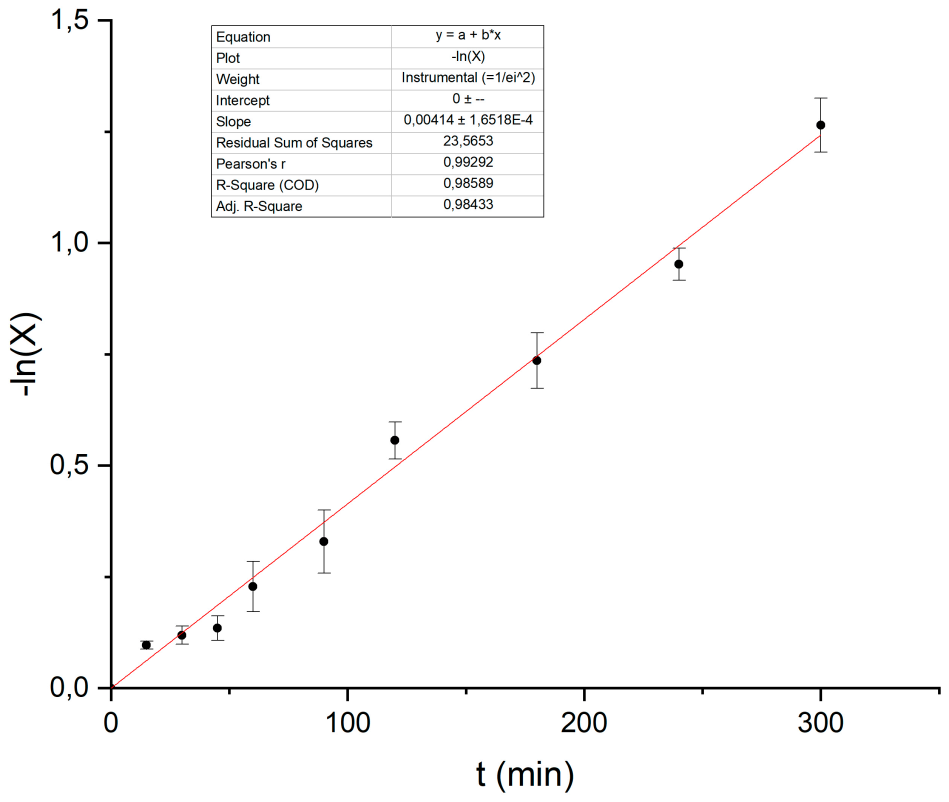

Back to Contents

Figure S11. HPLC trace for compound **3a**

Data File C:\Users\P...cuments\ChemStation\1\Data\Seq1 2022-09-09 22-13-29\005-P2-A8-545.D
Sample Name: 545

===

Acq. Operator : SYSTEM Seq. Line : 5
Sample Operator : SYSTEM
Acq. Instrument : LCMS Location : P2-A-08
Injection Date : 9/9/2022 11:38:16 PM Inj : 1
Inj Volume : 2.000 µl
Different Inj Volume from Sample Entry! Actual Inj Volume : 5.000 µl
Acq. Method : C:\Users\Public\Documents\ChemStation\1\Data\Seq1 2022-09-09 22-13-29\Purity_test450_SEQ.M
Last changed : 9/9/2022 10:13:26 PM by SYSTEM
Analysis Method : C:\Users\Public\Documents\ChemStation\1\Data\Seq1 2022-09-09 22-13-29\Purity_test450_SEQ.M (Sequence Method)
Last changed : 9/13/2022 3:37:42 PM by SYSTEM
(modified after loading)
Additional Info : Peak(s) manually integrated

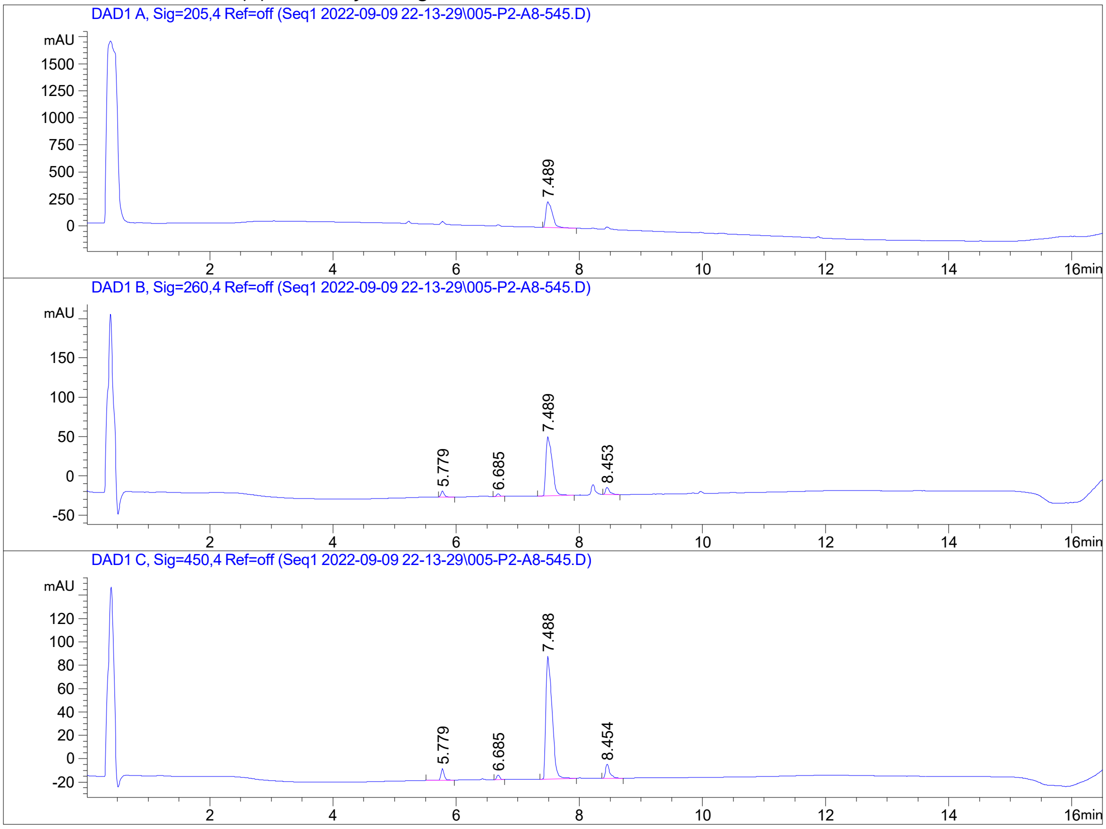

Back to Contents

Figure S12. HPLC trace for compound **3b**

```
Data File C:\Users\P...\ChemStation\1\Data\Seq1 2022-09-13 16-06-50\007-P1-A3-ARVI-20-16.D
Sample Name: ARVI-20-16

=====================================================================
Acq. Operator   : SYSTEM                        Seq. Line :   7
Sample Operator : SYSTEM
Acq. Instrument : LCMS                           Location :   P1-A-03
Injection Date  : 9/13/2022 5:59:42 PM                Inj :   1
                                               Inj Volume : 2.000 µl
Different Inj Volume from Sample Entry!  Actual Inj Volume : 20.000 µl
Acq. Method     : C:\Users\Public\Documents\ChemStation\1\Data\Seq1 2022-09-13 16-06-50
                  \Purity_test450_SEQ.M
Last changed    : 9/9/2022 10:13:26 PM by SYSTEM
Analysis Method : C:\Users\Public\Documents\ChemStation\1\Data\Seq1 2022-09-13 16-06-50
                  \Purity_test450_SEQ.M (Sequence Method)
Last changed    : 9/13/2022 6:37:22 PM by SYSTEM
                  (modified after loading)
Additional Info : Peak(s) manually integrated
```

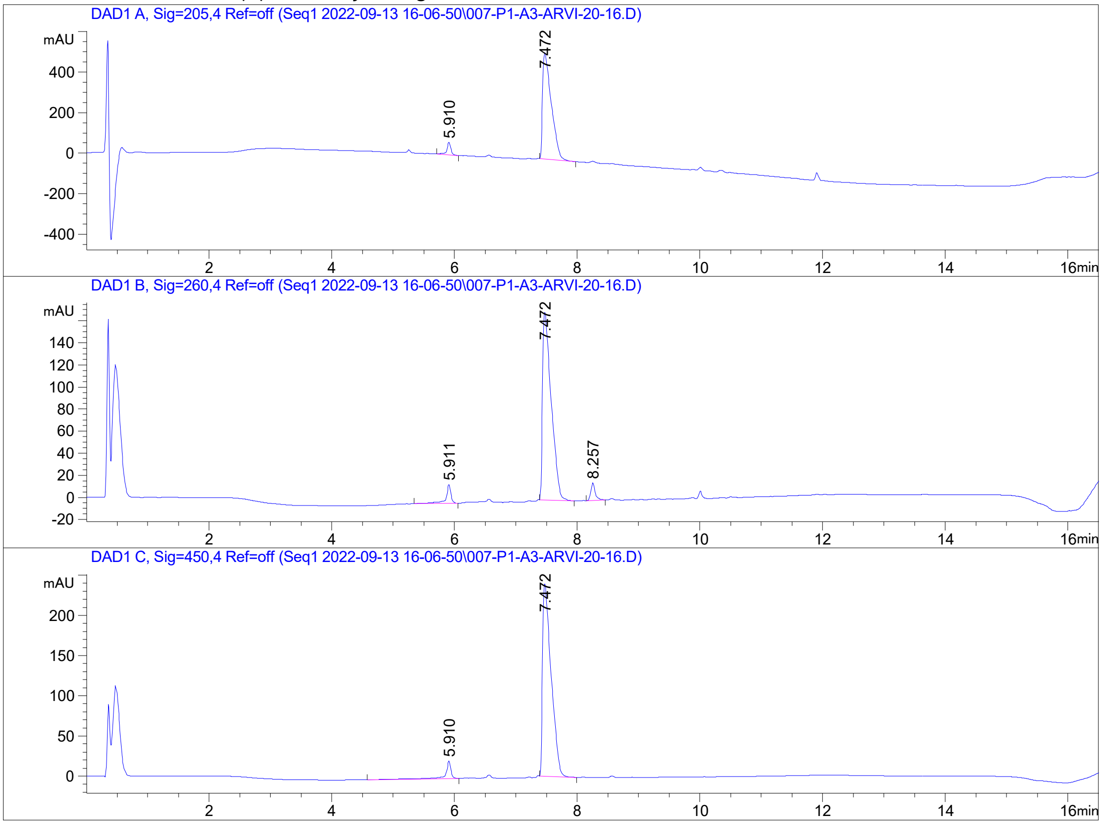

Figure S13. HPLC trace for compound **8**

```
Data File C:\Users\P...ments\ChemStation\1\Data\Seq1 2022-09-13 16-06-50\009-P1-A4-AR554.D
Sample Name: AR554

    =====================================================================
    Acq. Operator   : SYSTEM                          Seq. Line :   9
    Sample Operator : SYSTEM
    Acq. Instrument : LCMS                             Location :   P1-A-04
    Injection Date  : 9/13/2022 6:35:09 PM                  Inj :   1
                                                    Inj Volume : 2.000 µl
    Different Inj Volume from Sample Entry!  Actual Inj Volume : 10.000 µl
    Acq. Method     : C:\Users\Public\Documents\ChemStation\1\Data\Seq1 2022-09-13 16-06-50
                      \Purity_test450_SEQ.M
    Last changed    : 9/9/2022 10:13:26 PM by SYSTEM
    Analysis Method : C:\Users\Public\Documents\ChemStation\1\Data\Seq1 2022-09-13 16-06-50
                      \Purity_test450_SEQ.M (Sequence Method)
    Last changed    : 9/13/2022 6:53:31 PM by SYSTEM
                      (modified after loading)
    Additional Info : Peak(s) manually integrated
```

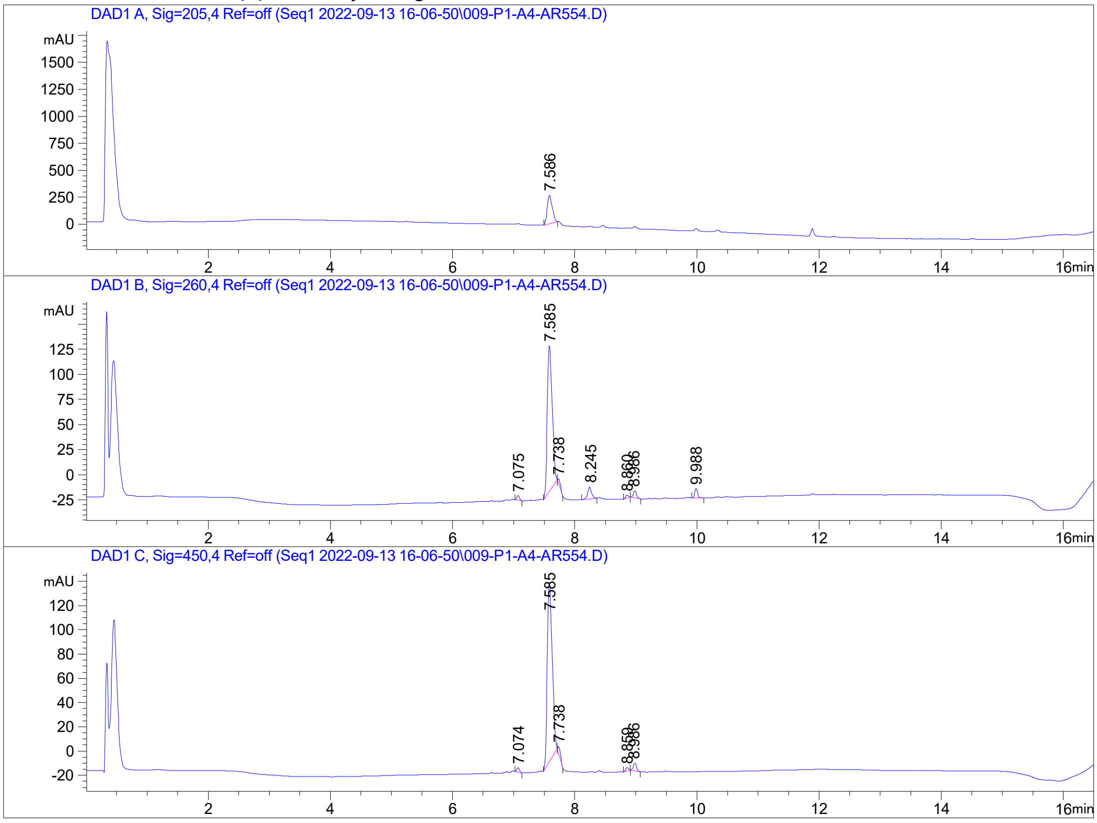

Back to Contents

Figure S14. HPLC trace for compound **10**

```
Data File C:\Users\P...ments\ChemStation\1\Data\Seq1 2022-09-13 16-06-50\005-P2-A5-AR542.D
Sample Name: AR542

    =====================================================================
    Acq. Operator   : SYSTEM                       Seq. Line :    5
    Sample Operator : SYSTEM
    Acq. Instrument : LCMS                          Location :   P2-A-05
    Injection Date  : 9/13/2022 5:22:10 PM               Inj :    1
                                                  Inj Volume : 2.000 µl
    Different Inj Volume from Sample Entry!  Actual Inj Volume : 5.000 µl
    Acq. Method     : C:\Users\Public\Documents\ChemStation\1\Data\Seq1 2022-09-13 16-06-50
                      \Purity_test530_SEQ.M
    Last changed    : 9/9/2022 10:05:11 PM by SYSTEM
    Analysis Method : C:\Users\Public\Documents\ChemStation\1\Data\Seq1 2022-09-13 16-06-50
                      \Purity_test530_SEQ.M (Sequence Method)
    Last changed    : 9/13/2022 5:44:50 PM by SYSTEM
                      (modified after loading)
    Additional Info : Peak(s) manually integrated
```

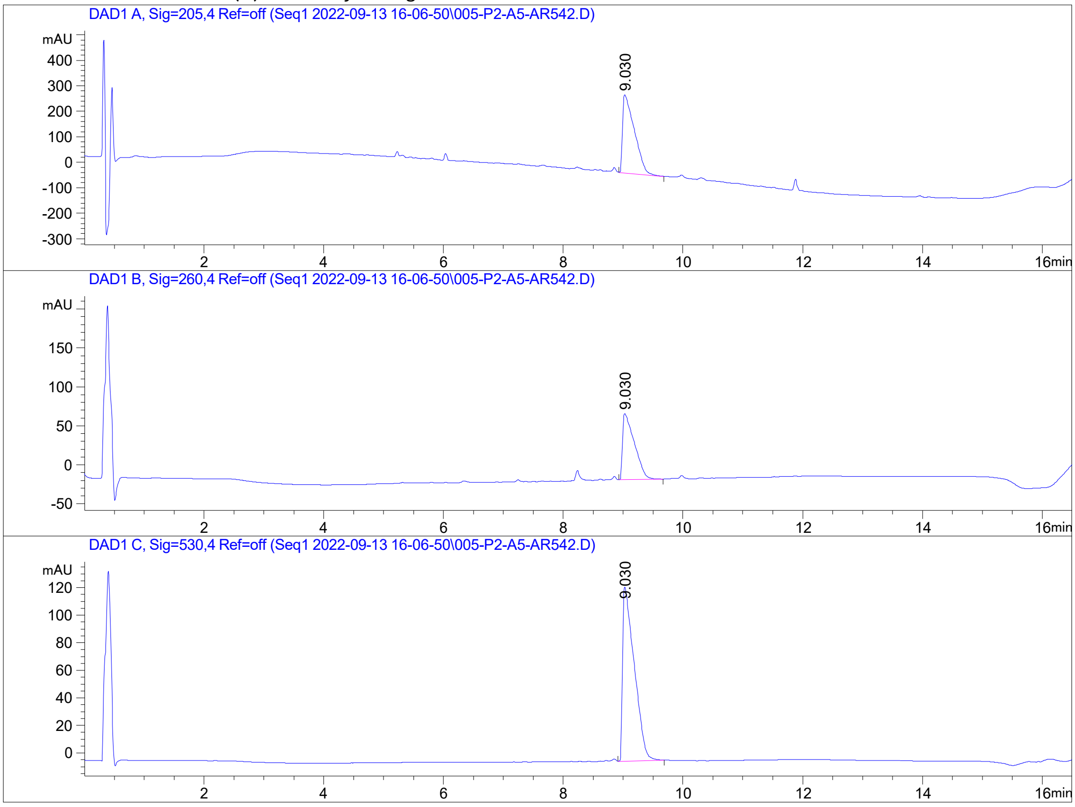

LCMS 9/13/2022 5:44:53 PM SYSTEM   Page   1 of 1

Back to Contents
